# TPRpred: a tool for prediction of TPR-, PPR- and SEL1-like repeats from protein sequences

**DOI:** 10.1186/1471-2105-8-2

**Published:** 2007-01-03

**Authors:** Manjunatha R Karpenahalli, Andrei N Lupas, Johannes Söding

**Affiliations:** 1 Department of Protein Evolution, Max-Planck-lnstitute for Developmental Biology, Spemannstrasse 35, D-72076 Tübingen, Germany

## Abstract

**Background:**

Solenoid repeat proteins of the Tetratrico Peptide Repeat (TPR) family are involved as scaffolds in a broad range of protein-protein interactions. Several resources are available for the prediction of TPRs, however, they often fail to detect divergent repeat units.

**Results:**

We have developed TPRpred, a profile-based method which uses a P-value-dependent score offset to include divergent repeat units and which exploits the tendency of repeats to occur in tandem. TPRpred detects not only TPR-like repeats, but also the related Pentatrico Peptide Repeats (PPRs) and SEL1-like repeats. The corresponding profiles were generated through iterative searches, by varying the threshold parameters for inclusion of repeat units into the profiles, and the best profiles were selected based on their performance on proteins of known structure. We benchmarked the performance of TPRpred in detecting TPR-containing proteins and in delineating the individual repeats therein, against currently available resources.

**Conclusion:**

TPRpred performs significantly better in detecting divergent repeats in TPR-containing proteins, and finds more individual repeats than the existing methods. The web server is available at , and the C++ and Perl sources of TPRpred along with the profiles can be downloaded from .

## Background

Solenoid repeat proteins have recently attracted interest because of their versatility as scaffolds for the engineering of protein-protein interactions [1]. This class of proteins is characterized by homologous, repeating structural units, which stack together to form an open-ended superhelical structure. Such an arrangement is in contrast to the structure of most proteins, which fold into a compact shape [2]. Solenoid structures adopt a variety of shapes, depending on the structural features of the repeating structural unit and the arrangement of individual units in the solenoid. The curvature created by the superhelical nature of these proteins predetermines the target proteins that can bind to them [3]. The Tetratrico Peptide Repeats (TPRs) together with their related repeats, the Pentatrico Peptide Repeats (PPRs) and the SEL1-like repeats, form a large family within the solenoid repeat proteins. The repeating unit of TPRs, PPRs and SEL1-like repeats are formed of two or more stacked 34, 35 and 36-amino acid *αα*-hairpin repeat units, respectively [4-6]. These solenoid repeat proteins are involved in a diverse spectrum of cellular functions such as cell cycle control, transcription, splicing, protein import, regulatory phosphate turnover and protein folding, by virtue of their tendency to bind target proteins [5,7,8].

Homologous structural repeat units are often highly divergent at the sequence level, a feature that makes their prediction challenging. Currently, several web-based resources are available for the detection of TPRs, including Pfam [9], SMART [10], and REP [11]. These resources use hidden Markov model (HMM) profiles or sequence profiles, which are constructed from the repeats trusted to belong to the family. However, the profiles used are constructed from closely homologous repeats; therefore, divergent repeat units often get a negative score and are not considered in computing the overall statistical significance, even though they are individually significant. For this reason Pfam, SMART, and REP perform with limited accuracy in detecting remote homologs of known TPR-containing proteins and in delineating the individual repeats within a protein [12,13]. For example, TPR-like repeats from the central domain of MalT protein [*E. coli*;PDB:1HZ4] are not detected by these resources. MalT is the transcription regulator of the maltose regulon, which is responsible for the uptake and catabolism of malto-oligosaccharides in *E. coli *[14]. In order to predict such divergent repeats, we have developed a specialized tool (TPRpred), which is able to predict TPR-, PPR- and SEL1-like repeats from protein sequences. The advantages of our method are the following:

• We construct optimized profiles through iterative searches by varying the threshold for inclusion of repeats into the profiles.

• We apply a score offset in such a way that repeats with P-value ≤ 0.01 will get a positive score. Therefore, even marginally significant repeats will contribute to the whole-protein P-value.

• Putative repeat units located near an already identified repeat get a tight-fit reward in order to account for the tendency of repeats to occur in tandem.

• Our tool reports not only P-values, based on the score distribution of true negatives derived from the known protein structures, but also computes a probability that a target sequence is a TPR protein.

## Implementation

Given a query sequence of length *L *and a sequence profile of length *W *representing a single repeat unit, TPRpred finds the best-scoring alignment of the sequence with an integer number of repeats, each of them aligned without internal gaps using standard log-odds scoring. Tandem repeats with a gap of ≤ *K *residues are rewarded with *r *bits, while gaps of > *K *residues are penalized with *g *bits (*K *= 10 and *g *= 0 in our benchmarks).

Since no internal gaps are allowed within repeats, the score distribution of the repeat profile with equal-length windows of unrelated sequences has an almost perfect Gaussian distribution. (The score is a sum of *W *independent random variables and therefore it approaches a Gaussian according to the central limit theorem.) The *σ *and *μ *parameters of this distribution are derived from a calibration search against a database of unrelated protein sequences from the SCOP database [15]. The tails of a Gaussian distribution approach zero much faster than the tails of a Gumbel distribution (which would be obtained if internal gaps were allowed). Therefore, the same positive score of a true repeat unit will generally have a much higher significance in the case of a Gaussian as compared to a Gumbel distribution. Hence, the restriction of ungapped repeats increases the sensitivity of TPRpred for detecting ungapped repeat families such as TPR-, PPR- and, SEL1-like repeat proteins and others with duplicated helical hairpins.

If the reward *r *for closely spaced repeat units is set low (e.g. zero) then one will fail to detect many repeats if their score is below zero. This is the case for the HMMER software [16], where often repeat instances have scores below zero even though their P-values are significant (e.g. below 0.01). Since alignment algorithms find the alignment with maximum score, they will skip repeat instances that are assigned negative scores. On the other hand, if *r *is set high, many false positive repeat units will be found within *K *residues of an already ascertained repeat unit. We therefore set the reward *r *such that the probability of finding a false positive repeat instance within *K *residues of another repeat is *p*_*r *_= 0.01. In the appendix, it is shown that this requires to set the tight fit reward *r *to

r=−2σ×erfc−1[2(1−(1−pr)1/K)]−μ.     (1)
 MathType@MTEF@5@5@+=feaafiart1ev1aaatCvAUfKttLearuWrP9MDH5MBPbIqV92AaeXatLxBI9gBaebbnrfifHhDYfgasaacH8akY=wiFfYdH8Gipec8Eeeu0xXdbba9frFj0=OqFfea0dXdd9vqai=hGuQ8kuc9pgc9s8qqaq=dirpe0xb9q8qiLsFr0=vr0=vr0dc8meaabaqaciaacaGaaeqabaqabeGadaaakeaacqWGYbGCcqGH9aqpcqGHsisldaGcaaqaaiabikdaYaWcbeaaiiGakiab=n8aZjabgEna0kabbwgaLjabbkhaYjabbAgaMjabbogaJnaaCaaaleqabaGaeyOeI0IaeGymaedaaOWaamWaaeaacqaIYaGmdaqadaqaaiabigdaXiabgkHiTiabcIcaOiabigdaXiabgkHiTiabdchaWnaaBaaaleaacqWGYbGCaeqaaOGaeiykaKYaaWbaaSqabeaacqaIXaqmcqGGVaWlcqWGlbWsaaaakiaawIcacaGLPaaaaiaawUfacaGLDbaacqGHsislcqWF8oqBcqGGUaGlcaWLjaGaaCzcamaabmaabaGaeGymaedacaGLOaGaayzkaaaaaa@53C4@

Here erfc^-1 ^is the inverse of the complementary error function, and *σ *and *μ *are derived from the calibration of the profile as explained before.

To further increase sensitivity, we add an offset *c *to the repeat unit match score in such a way that the probability for the observation of a repeat in an unrelated database protein of length *L *is equal to *p*_*c *_= 0.01. In the appendix it is shown that this requires to set the offset *c *to

c=−2σ×erfc−1[2(1−(1−pc)1/(L−W+1))]−μ.     (2)
 MathType@MTEF@5@5@+=feaafiart1ev1aaatCvAUfKttLearuWrP9MDH5MBPbIqV92AaeXatLxBI9gBaebbnrfifHhDYfgasaacH8akY=wiFfYdH8Gipec8Eeeu0xXdbba9frFj0=OqFfea0dXdd9vqai=hGuQ8kuc9pgc9s8qqaq=dirpe0xb9q8qiLsFr0=vr0=vr0dc8meaabaqaciaacaGaaeqabaqabeGadaaakeaacqWGJbWycqGH9aqpcqGHsisldaGcaaqaaiabikdaYaWcbeaaiiGakiab=n8aZjabgEna0kabbwgaLjabbkhaYjabbAgaMjabbogaJnaaCaaaleqabaGaeyOeI0IaeGymaedaaOWaamWaaeaacqaIYaGmdaqadaqaaiabigdaXiabgkHiTiabcIcaOiabigdaXiabgkHiTiabdchaWnaaBaaaleaacqWGJbWyaeqaaOGaeiykaKYaaWbaaSqabeaacqaIXaqmcqGGVaWlcqGGOaakcqWGmbatcqGHsislcqWGxbWvcqGHRaWkcqaIXaqmcqGGPaqkaaaakiaawIcacaGLPaaaaiaawUfacaGLDbaacqGHsislcqWF8oqBcqGGUaGlcaWLjaGaaCzcamaabmaabaGaeGOmaidacaGLOaGaayzkaaaaaa@5934@

This ensures that even repeat units with no neighbours within *K *residues will get detected, if their P-value is better than 0.01, independent of the original score baseline (which depends on a null model that is not appropriate for this purpose). At the same time, this global offset guarantees that only very rarely (with probability ≈ 10^-4^) TPRpred will find more than one false positive repeat unit in an unrelated protein. TPRpred not only computes P-values, which are solely based on the true negative score distribution, but is also able to report the probability that a target sequence is a true homolog, by making use of both the true positive and true negative score distributions. In addition, TPRpred is able to calculate more realistic (i.e. less optimistic) E-values, by calibrating with true negative sequences as opposed to random sequences. The algorithm has been implemented as a computer program "TPRpred", written in C++ (a Perl version is also available). The profiles used by TPRpred are generated by the program ppmake in the TPRpred software package. The Henikoff and Henikoff sequence weighting and pseudocounts are added in a way completely analogous to the procedure used in PSI-BLAST software package [17], except that the Gonnet matrix is used instead of BLOSUM62. The tool has been tested on a GNU/Linux platform with a i386 processor architecture.

## Results and discussion

### Definition of TPR-like and non-TPR-like proteins

We define the positive (i.e. the TPR-like) and the negative (i.e. non-TPR-like) set of protein sequences by reference to a set of 13 *bona fide *TPR-like domains. These are the domains contained in the "TPR-like" superfamily [a.118.8] of the SCOP database (version 1.69) [15], which consists of the TPR family and the MalT protein. (We use a SCOP version filtered to 70% maximum pairwise sequence identity, available from the ASTRAL server [18].) The SCOP classification of MalT as TPR-like is supported both by structural and sequence similarity: (1) A DALI search [19] with the MalT structure [PDB:1HZ4] for structural neighbors yields ten SCOP domains above Z-score of 10, all of them from the TPR family in SCOP (supplementary material, see the file "Additional File 1"). (2) Furthermore, a search with the remote homology prediction server HHpred [20,21] through the SCOP database readily yields TPRs as closest relatives (supplementary material, see the file "Additional File 1"). To take into account more recent TPR structures not yet contained in SCOP v1.69, we used DALI to search the PDB database (version of December 2005) with the 13 *bona fide *TPR-like repeat domains as defined by SCOP. We included all structures into our true positve set that obtained a Z-score of at least 10 with one or more of the *bona fide *TPR-like repeat domains.

The true negative is defined conservatively to include all sequences in SCOP vl.69 (filtered to 70%) which have no Z-score better than 5 with any of the 13 *bona fide *TPR-like repeat domains (supplementary material, see the file "Additional File 2"). This ensures that marginal cases of proteins which can be neither classified safely as TPR-like nor as non-TPR-like will be ignored in the benchmark.

### Profile generation and test set

The performance in the high-selectivity regime of sequence profiles depends on the number of close homologs, whereas the performance in the high-sensitivity regime depends on the number of remote homologs used in constructing the profiles. Relaxing the threshold value to include remote homologs often results in false positives. To optimize the trade-off between remote homologs and false positives, we have constructed a series of TPR profiles. These profiles were generated by iterative searches against the non-redundant (NR) database at NCBI, filtered to a maximum pairwise sequence identity of 70% (NR-70) by CD-HIT [22,23]. Prior to the searches we broadly removed homologs of MalT [GI:16131294], which we intended to use as a test set, from the NR-70 database using three iterations of PSI-BLAST [17] at an E-value cutoff of 1.

Homologs of MalT contain divergent TPR units and therefore represent a challenging test set. These proteins belong to the STAND family of ATPases [24,25], which themselves are part of the AAA+ superfamily [26]. We extracted these sequences conservatively with PSI-BLAST (two iterations, E-value cutoff of 10^-4^) from NR-70, using the central domain of MalT [GI:17942835] as a query sequence. Using the defining characteristic of STAND proteins, namely an N-terminal P-loop NTPase domain, as a criterion we selected 56 proteins for the test set. The sequences of these proteins are given in the supplementary material (see the file "Additional File 3").

**Figure 1:**
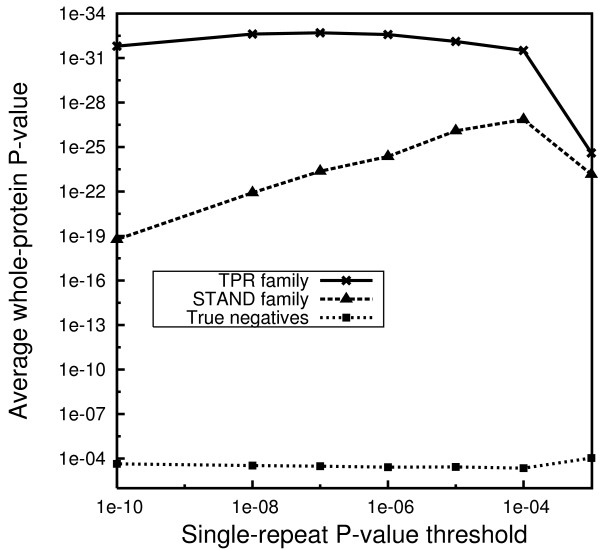
**Selection of the best TPR profile**. The geometric average of the whole-protein P-value for the top 10 hits in each test set is plotted against the profile's single-repeat P-value threshold. The profile obtained for a single-repeat P-value threshold of 10^-4 ^was selected as best.

We performed iterative searches to convergence on NR-70 minus STAND proteins with various threshold parameters (whole-protein E-value, and single-repeat P-value). The initial searches were seeded with a manually prepared structure-based sequence alignment of known TPR protein structures (supplementary material, see the file "Additional File 4"). We tested the resulting profiles on the STAND family, TPR family, and the true negative set. The best profile was selected based on its performance on the STAND family, as illustrated in Figure 1.

Further, we built the PPR and the SEL1-like profiles by using the same procedure and cutoff value as for the TPR profile.

### Benchmarking

We benchmarked our method and the web-server against Pfam, SMART and REP.

#### Comparison of TPRpred and HMMER

**Figure 2:**
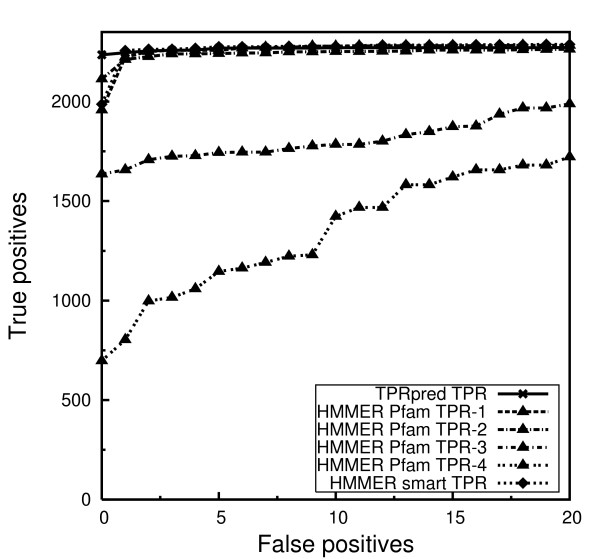
**ROC plot comparing the performance of TPRpred and HMMER**. Sensitivity of the methods, measured by the number of true positives detected at varying numbers of false positives.

To demonstrate the sensitivity/selectivity of TPRpred against HMMER (version 2.3, default parameters), which is the underlying method employed by the Pfam and SMART web-servers, we benchmarked the performance of both these methods, and the results are shown using the receiver operating characteristic (ROC) plot as illustrated in Figure 2. We could not benchmark against REP, because the stand-alone version is not available. The data sets for the benchmark were obtained using the same true positive and true negative sets which we defined in the profile generation section, but with a 25% maximum sequence identity. In order to enrich these data sets with reliable homologs, two iterations of PSI-BLAST searches were performed for each domain sequence. The first iteration was performed on the NR-90 database. The hits with an E-value ≤ 10^-3 ^and ≥ 85% coverage to the query sequence were extracted into a multiple alignment, that was used to jump-start the second iteration against the NR-70 database. The same selection criteria as in the first iteration were applied in obtaining the homologs for the query. The resulting enriched data sets were simultaneously filtered to a 50% maximum sequence identity using CD-HIT to reduce the redundancy.

Both methods were used to perform searches through the true positive and true negative data sets, using their own TPR profiles or HMMs. The ROC plot shows that TPRpred detects more sequences with E-value better than the first false positive compared to HMMER. However, for lower selectivity TPRpred performance is comparable to HMMER.

#### Comparison of the web-servers using STAND family members

To assess the sensitivity of TPRpred in detecting divergent TPR units over Pfam (version 20.0 of May 2006), SMART (5.0), and REP (1.1), we evaluated the performance of the web-servers using the STAND family test set. Additionally, we also used 53 true negative sequences by selecting arbitrarily from the all-*α *class of the SCOP database. The sequences of these proteins are given in the supplementary material (see the file "Additional File 5"). The hits that were confidently predicted according to the web-servers for the STAND proteins are tabulated in Table 1. None of the servers detected false positives from the true negative sequences (data not shown). This shows that all the servers are unbiased to the *α*-helical proteins which are unrelated.

**Table 1:** Comparison of the results obtained from the web-servers using a set of 56 STAND family members.

	**TPRpred**	**Pfam**	**SMART**	**REP**
Proteins detected (% of total)	48 (85%)	24 (42%)	6 (10%)	5 (8%)
Individual repeats detected	302	50	30	35

TPRpred performs significantly better in detecting the TPR units from the members of the STAND family, although sequences of the STAND family members were explicitly excluded from our TPR profile. For instance, the 8 TPR units present in MalT [12] were detected only by our server. Overall, TPRpred detected twice as many proteins as TPR-containing proteins and over 6 fold more individual repeats as the next best web-server, Pfam. This could be due to the more sensitive Gaussian scoring as well as the score base-line strategy employed by our tool.

#### Comparison of the web-servers using known protein structures

**Figure 3:**
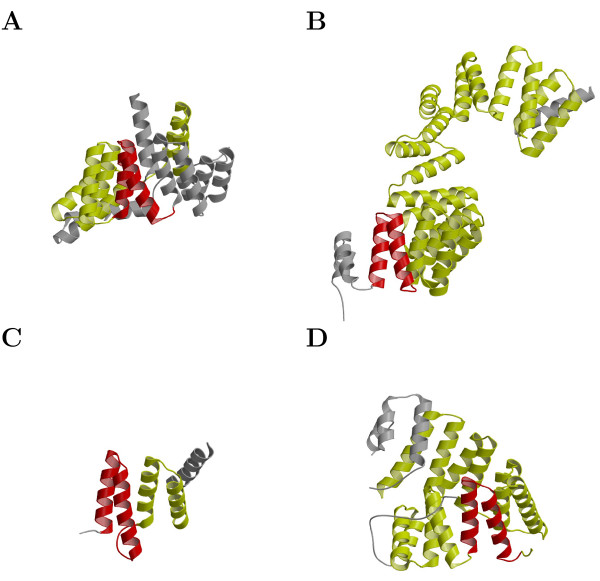
**The accuracy of TPRpred in detecting individual repeats**. The TPRs detected only by TPRpred are shown in red, whereas TPRs also detected by the other servers are shown in yellow, and the remaining residues are shown in grey. Structures in which all TPRs are only recognized by TPRpred are omitted. (A) *E. coli *NlpI [PDB:1XNF, chain A]. (B) Human N-acetylglucosamine transferase, TPR domain [PDB:1W3B, chain A]. (C) Peptide-substrate-binding domain of human type I collagen prolyl 4-hydroxylase [PDB:1TJC, chain A]. (D) Human PEX5 [PDB:1FCH, chain A]. The figure was generated using MOLSCRIPT [28] and Raster3D [29].

In order to assess the sensitivity of the web-servers in detecting the individual repeat units, we submitted the sequences of the TPR structure set, along with 2 SEL1-like repeat proteins classified under the HCP-like family [SCOP:a.118.18.1], to TPRpred, Pfam, SMART, and REP web-servers. The number of repeats detected confidently for each sequence are tabulated in Table 2 and the repeats detected only by TPRpred are shown in Figure 3. The TPR structure set contains both proteins that were present in the training databases of the individual methods (Table 2, top) and proteins whose structure became available subsequently (Table 2, bottom). All servers performed well on the former proteins, although TPRpred stood out with 100% detected individual repeats over the other servers, which only detected between 70% and 90%, but the real difference between servers became visible on the latter proteins. Here, TPRpred recognized all proteins as TPR-containing, whereas the other servers recognized less than half, and TPRpred detected 97% of individual repeats, whereas the other servers detected only about 54%.

**Table 2:** The comparison of the results obtained from the web-servers using known structures

**PDB-ID**	**Name**	**Repeat Type**	**Actual Repeats**	**TPRpred**	**Pfam**	**SMART**	**REP**
**Structures used in profile generation by TPRpred**							
1A17	Protein phosphatase 5	TPR	3	3	3	3	0
1KT1	Fkbp51	TPR	3	3	2	2	3
1ELR	Hop(TPR2a domain)	TPR	3	3	3	3	3
1IHG	Cyclophilin 40	TPR	3	3	3	3	3
1ELW	Hop (TPR1 domain)	TPR	3	3	3	3	3
1HH8	P67phox	TPR	3	3	3	3	3
1FCH*	PEX5 (Human)	TPR	7	7	4	4	6
1HXI	Pex5 (Trypanosoma)	TPR	3	3	3	3	3
1KLX	Hcpb	SEL1	3	3	3	3	3
1OUV	Hcpc	1^†^+6^‡^	7	1^†^+6^‡^	1^†^+6^‡^	7^‡^	7^†^

**Total**			**38**	**38**	**34**	**33**	**27**
**% of total**				**100%**	**89%**	**86%**	**71%**

**Structures not used in profile generation by TPRpred**							
1P5Q	Fkbp52	TPR	3	3	3	3	3
2C2L	CHIP	TPR	3	3	3	3	3
1XNF*	Nlpi	TPR	4	4	3	3	3
1W3B*	GlcNAc transferase	TPR	10	10	9	9	9
1TJC*	Collagen Hydroxylase	TPR	2	2	1	1	0
1HZ4	MalT	TPR	8	8	0	0	0
1NZN	Fis1	TPR	2	1	0	0	0
1ZU2	Tom20(Plant)	TPR	2	2	0	0	0
1ZBP	VPA1032	TPR	1	1	0	0	0

**Total**			**35**	**34**	**19**	**19**	**18**
**% of total**				**97%**	**54%**	**54%**	**51%**

* Structures shown in Figure 3

^† ^TPR

^‡ ^SEL1-like repeat

See also Figure 3. The actual number of repeats for each entry and the number of repeats detected by various web-servers are tabulated.

#### Comparison of TPRpred, Pfam and SMART on the human proteome

To assess the global gain in the protein annotation of TPRpred over Pfam and SMART, we scanned a set of 37 444 sequences of the human proteome downloaded from Integr8 [27]. The number of proteins and individual repeats detected confidently by TPRpred, Pfam and SMART are tabulated in Table 3. TPRpred detected more proteins as TPR-containing proteins and over 2 fold more individual repeats than Pfam and SMART.

**Table 3:** Comparison of the results obtained from TPRpred, Pfam and SMART using a set of 37444 sequences of the human proteome.

	**TPRpred**	**Pfam**	**SMART**
Proteins detected	326	262	149
Individual repeats detected	1505	725	746

## Conclusion

TPRpred is a profile-sequence comparison method for predicting solenoid repeat proteins of TPRs, PPRs and SEL1-like repeats. It shows a marked improvement over existing methods, particularly in the detection of non-canonical, divergent repeats. We attribute this to the exploitation of simple traits such as the tendency of repeats to occur in tandem, robust statistical evaluations and the construction of profiles by iterative searches. The algorithmic improvements of the P-value-dependent score offset as well as the tight-fit reward are quite general and easily transferable to other repeat detection approaches.

## Availability and requirements

• **Project name: **TPRpred.

• **Project home page: **

• **Sources: **The C++ and Perl source codes for TPRpred along with the profiles are freely available by anonymous ftp to 

• **Operating systems: **Linux, Unix.

• **Programming language: **C++ and PERL.

• **Other requirements: **The Perl script requires Perl interpreter version 5.8.5 or higher.

• **License: **GNU GENERAL PUBLIC LICENSE 

• **Any restrictions to use by non-academics: **None.

## Authors' contributions

JS developed the algorithm and programmed the Perl version. MRK was involved in the analysis and interpretation of the data, wrote the wrapper program for the web-interface and drafted the manuscript. ANL supervised the overall work. ANL and JS critically revised the manuscript. All authors read and approved the final manuscript.

## Appendix

First we show that, if the tight-fit reward *r *is calculated according to equation 1, the P-value to observe a second repeat unit within *K *residues from an existing one will be *p*_*r*_. To start, note that the P-value for observing a score *S *> *s *between the profile and an unrelated equal-length sequence window is

Prob(S>s)=∫s∞1/2πσ2exp⁡((S−μ)22σ2)dS=12erfc(S−μ2σ),
 MathType@MTEF@5@5@+=feaafiart1ev1aaatCvAUfKttLearuWrP9MDH5MBPbIqV92AaeXatLxBI9gBaebbnrfifHhDYfgasaacH8akY=wiFfYdH8Gipec8Eeeu0xXdbba9frFj0=OqFfea0dXdd9vqai=hGuQ8kuc9pgc9s8qqaq=dirpe0xb9q8qiLsFr0=vr0=vr0dc8meaabaqaciaacaGaaeqabaqabeGadaaakeaacqqGqbaucqqGYbGCcqqGVbWBcqqGIbGycqGGOaakcqWGtbWucqGH+aGpcqWGZbWCcqGGPaqkcqGH9aqpdaWdXaqaaiabigdaXiabc+caVmaakaaabaGaeGOmaidcciGae8hWdaNae83Wdm3aaWbaaSqabeaacqaIYaGmaaaabeaaaeaacqWGZbWCaeaacqGHEisPa0Gaey4kIipakiGbcwgaLjabcIha4jabcchaWnaabmaabaWaaSaaaeaacqGGOaakcqWGtbWucqGHsislcqWF8oqBcqGGPaqkdaahaaWcbeqaaiabikdaYaaaaOqaaiabikdaYiab=n8aZnaaCaaaleqabaGaeGOmaidaaaaaaOGaayjkaiaawMcaaiabdsgaKjabdofatjabg2da9maalaaabaGaeGymaedabaGaeGOmaidaaiabbwgaLjabbkhaYjabbAgaMjabbogaJnaabmaabaWaaSaaaeaacqWGtbWucqGHsislcqWF8oqBaeaadaGcaaqaaiabikdaYaWcbeaakiab=n8aZbaaaiaawIcacaGLPaaacqGGSaalaaa@690A@

where erfc() is the complementary error function. Because the alignment between the profile and equal-length sequence windows is gap-free, the scores of neighbouring sequence windows can be assumed to be independent from each other. Hence, by elementary probability theory, the probability to obtain a score *S*_*i *_+ *r *larger than zero at any of *K *start positions (*i *= 1,...,*K*) is

Prob(S1+r>0 or ... or SK+r>0)=1−Prob(S1+r≤0 and ... and SK+r≤0)=1−∏i=1K(1−Prob(Si>−r))=1−(1−12erfc(−r−μ2σ))K.
MathType@MTEF@5@5@+=feaafiart1ev1aaatCvAUfKttLearuWrP9MDH5MBPbIqV92AaeXatLxBI9gBaebbnrfifHhDYfgasaacH8akY=wiFfYdH8Gipec8Eeeu0xXdbba9frFj0=OqFfea0dXdd9vqai=hGuQ8kuc9pgc9s8qqaq=dirpe0xb9q8qiLsFr0=vr0=vr0dc8meaabaqaciaacaGaaeqabaqabeGadaaakeaafaqaaeWadaaabaGaeeiuaaLaeeOCaiNaee4Ba8MaeeOyaiMaeiikaGIaem4uam1aaSbaaSqaaiabigdaXaqabaGccqGHRaWkcqWGYbGCcqGH+aGpcqaIWaamcqqGGaaicqqGVbWBcqqGYbGCcqqGGaaicqGGUaGlcqGGUaGlcqGGUaGlcqqGGaaicqqGVbWBcqqGYbGCcqqGGaaicqWGtbWudaWgaaWcbaGaem4saSeabeaakiabgUcaRiabdkhaYjabg6da+iabicdaWiabcMcaPaqaaiabg2da9aqaaiabigdaXiabgkHiTiabbcfaqjabbkhaYjabb+gaVjabbkgaIjabcIcaOiabdofatnaaBaaaleaacqaIXaqmaeqaaOGaey4kaSIaemOCaiNaeyizImQaeGimaaJaeeiiaaIaeeyyaeMaeeOBa4MaeeizaqMaeeiiaaIaeiOla4IaeiOla4IaeiOla4IaeeiiaaIaeeyyaeMaeeOBa4MaeeizaqMaeeiiaaIaem4uam1aaSbaaSqaaiabdUealbqabaGccqGHRaWkcqWGYbGCcqGHKjYOcqaIWaamcqGGPaqkaeaaaeaacqGH9aqpaeaacqaIXaqmcqGHsisldaqeWaqaaiabcIcaOiabigdaXiabgkHiTiabbcfaqjabbkhaYjabb+gaVjabbkgaIjabcIcaOiabdofatnaaBaaaleaacqWGPbqAaeqaaOGaeyOpa4JaeyOeI0IaemOCaiNaeiykaKIaeiykaKcaleaacqWGPbqAcqGH9aqpcqaIXaqmaeaacqWGlbWsa0Gaey4dIunaaOqaaaqaaiabg2da9aqaaiabigdaXiabgkHiTmaabmaabaGaeGymaeJaeyOeI0YaaSaaaeaacqaIXaqmaeaacqaIYaGmaaGaeeyzauMaeeOCaiNaeeOzayMaee4yam2aaeWaaeaadaWcaaqaaiabgkHiTiabdkhaYjabgkHiTGGaciab=X7aTbqaamaakaaabaGaeGOmaidaleqaaOGae83WdmhaaaGaayjkaiaawMcaaaGaayjkaiaawMcaamaaCaaaleqabaGaem4saSeaaOGaeiOla4caaaaa@A609@

We now set this expression to *p*_*r*_, the P-value for observing a spurious second repeat within *K *residues of an already detected one. Solving for *r *yields equation 1.

Equation 2 can be proved analogously. A database protein of length *L *contains *L *- *W *+ 1 windows of length *W*. The score between the profile and the *i'th *window is written as *S*_*i *_+ *c*, which already includes the score offset *c *that needs to be determined. The probality that at least one of the scores is larger than zero is the same as in the previous equation when *r *is replaced by *c*, and *K *by *L *- *W *+ 1. Setting the right-hand expression equal to *p*_*c *_and solving for *c *then yields equation 2.

## Supplementary Material

Additional File 1 **Relatives of MalT by structure and sequence comparison**. DALI and HHpred search results for MalT [PDB:1HZ4] Click here for file

Additional File 2 **Structural neighbours of TPRs**. Structural neighbours of known TPRs according to the DALI structure comparison server. The structures with Z scores ≥ 5 are tabulated. The PDB codes were mapped on to the SCOP domain database. Click here for file

Additional File 3 **STAND proteins**. The set of 56 STAND family members. Click here for file

Additional File 4 **Structure-based sequence alignments**. Structure-based sequence alignments for TPR and SEL1-like repeat families. Click here for file

Additional File 5 **True negative data set used in servers benchmarking**. Arbitrarily selected 53 true negative sequences from the all-*α *class of the SCOP database. Click here for file
