# Supplementary material for: TPRpred: a tool for prediction of TPR-, PPR- and SEL1-like repeats from protein sequences
Source: BMC Bioinformatics. 2007 Jan 3;8:2. doi: 10.1186/1471-2105-8-2 (PMC1774580; doi:10.1186/1471-2105-8-2)
Supplement: Additional File 1 — Relatives of MalT by structure and sequence comparison. DALI and HHpred search results for MalT [PDB:1HZ4] [file 1471-2105-8-2-S1.PDF]

DALI search for structural neighbors of MalT 1HZ4_A

1hz4A: Structural neighbours in PDB90 and structural alignments

* PDB90 is a representative subset of PDB chains that are less than 90 % sequence identical to each other
* No: the top 20 alignments, sorted by Z-score, are shown
* Chain: PDB entry code plus chain identifier
* raw-score: the sum of weighted similarities of intra molecular distances that Dali maximizes
* Z-score: normalized score that depends on the size of the structures
* %id: percentage of identical amino acids over all structurally equivalent residues
* lali: number of structurally equivalent residues
* rmsd: root-mean-square deviation of C-alpha atoms in the least-squares superimposition of the structurally equivalent C-alpha atoms
* Description: the COMPND record from the PDB entry

No	Chain	SCOP family	raw-score	Z-score	%id	lali	rmsd	Description
1	1hz4A	a.118.8.2	6690.2	62.3	100	366	0.0	MALT REGULATORY
2	1w3bA	a.118.8.1	1773.5	14.8	15	219	3.2	UDP-N-ACETYLGLU
3	1fchA	a.118.8.1	1628.8	14.1	11	231	4.9	PEROXISOMAL TA
4	1qqeA	a.118.8.1	1582.3	13.9	11	234	9.5	VESICULAR TRAN
5	1xnfB	a.118.8.1	1495.3	13.3	12	228	5.2	LIPOPROT
6	1elrA	a.118.8.1	891.7	12.4	10	124	2.9	TPR2A-DOMAIN OF
7	1a17	a.118.8.1	877.1	11.9	11	136	6.8	SERINE/THR
8	1kt0A	a.118.8.1	918.1	11.7	9	154	13.1	51 KDA FK506-BI
9	1elwA	a.118.8.1	808.5	11.5	8	115	2.6	TPR1-DOMAIN OF
10	1hh8A	a.118.8.1	1160.8	11.3	10	157	3.6	NEUTROPHIL CYT
11	1na0A	a.118.8.1	778.7	11.3	15	113	0.0	DESIGNED PROTE
12	1ihgA	a.118.8.1	1015.3	10.6	9	162	10.7	CYCLOPHILIN 40
13	1kt1A	a.118.8.1	1065.1	10.5	10	153	8.1	FK506-BINDING P
14	1tjcA	a.118.8.1	547.6	10.3	14	89	2.5	PROLYL 4-
15	1qjbB	a.118.7.1	1144.6	9.8	10	170	3.8	14-3-3 PROTEIN
16	1o9fA	-	1121.0	9.7	5	171	3.4	14-3-3-LIKE PR
17	1ywtA	-	900.7	9.2	6	157	4.4	14-3-3 PROTEIN
18	1iygA	a.118.8.1	605.5	8.1	8	112	4.7	HYPOTHETICAL PR
19	1fp3A	a.102.1.3	1095.6	8.1	9	211	4.7	N-ACYL-D-GLUCO
20	1hxiA	a.118.8.1	450.6	7.8	8	95	8.9	PEROXISOME TAR

HHpred sequence search for distant relatives of MalT 1HZ4_A

PSI-BLAST rounds: 3
Query d1hz4a_ a.118.8.2 (A:) Transcription factor MalT domain III {Escherichia coli}

No	Hit		Prob	E-value	P-value	Score
1	d1hz4a_	a.118.8.2	100.0	0	0	438.7
2	d1hz4a_	a.118.8.2	99.9	2.5E-25	2.5E-29	199.3
3	d1fcha_	a.118.8.1	99.7	7.6E-19	7.9E-23	151.9
4	d1w3ba_	a.118.8.1	99.3	1.1E-14	1.1E-18	121.5
5	d1fcha_	a.118.8.1	99.1	3.8E-15	3.9E-19	124.8
6	d1qqea_	a.118.8.1	99.1	2.8E-14	3E-18	118.4
7	d1qqea_	a.118.8.1	98.7	2.6E-13	2.7E-17	111.4
8	d1ld8a_	a.118.6.1	98.5	1.6E-12	1.7E-16	105.6
9	d1ld8a_	a.118.6.1	98.4	2.4E-13	2.5E-17	111.6
10	d1w3ba_	a.118.8.1	98.2	7.5E-11	7.7E-15	93.4
