# Supplementary material for: TPRpred: a tool for prediction of TPR-, PPR- and SEL1-like repeats from protein sequences
Source: BMC Bioinformatics. 2007 Jan 3;8:2. doi: 10.1186/1471-2105-8-2 (PMC1774580; doi:10.1186/1471-2105-8-2)
Supplement: Additional File 2 — Structural neighbours of TPRs. Structural neighbours of known TPRs according to the DALI structure comparison server. The structures with Z scores ≥ 5 are tabulated. The PDB codes were mapped on to the SCOP domain database. [file 1471-2105-8-2-S2.PDF]

>d1qoja_ a.2.9.1 (A:) C-terminal UvrC-binding domain of UvrB {Escherichia coli}
>d1cuna1 a.7.1.1 (A:7-115) Spectrin alpha chain {Chicken (Gallus gallus)}
>d1owaa_ a.7.1.1 (A:) Spectrin alpha chain {Human (Homo sapiens)}
>d1qlaa1 a.7.3.1 (A:458-655) Fumarate reductase {Wolinella succinogenes}
>d1hx1b_ a.7.7.1 (B:) BAG-family molecular chaperon regulator-1, BAG1 {Human (Homo sapiens)}
>d1m62a_ a.7.7.1 (A:) Silencer of death domains, Sodd (Bag4) {Human (Homo sapiens)}
>d1fjgt_ a.7.6.1 (T:) Ribosomal protein S20 {Thermus thermophilus}
>d1sumb_ a.7.12.1 (B:) PhoU homolog TM1734 {Thermotoga maritima}
>d1a32__ a.16.1.2 (-) Ribosomal protein S15 {Bacillus stearothermophilus}
>d1mtyg_ a.23.3.1 (G:) Methane monooxygenase hydrolase, gamma subunit {Methylococcus capsulatus}
>d256ba_ a.24.3.1 (A:) Cytochrome b562 {Escherichia coli}
>d1nzea_ a.24.18.1 (A:) Oxygen-evolving enhancer protein 3, {Spinach (Spinacia oleracea)}
>d2a0b__ a.24.10.1 (-) Aerobic respiration control sensor protein, ArcB {Escherichia coli}
>d1ug7a_ a.24.24.1 (A:) Domain from hypothetical 2610208m17rik protein {Mouse (Mus musculus)}
>d1ile_1 a.27.1.1 (642-821) Isoleucyl-tRNA synthetase (IleRS) {Thermus thermophilus}
>d1h3na1 a.27.1.1 (A:687-814) Leucyl-tRNA synthetase (LeuRS) {Thermus thermophilus}
>d1rj1a_ a.29.6.1 (A:) Invertase inhibitor {Common tobacco (Nicotiana tabacum)}
>d1bg1a1 a.47.1.1 (A:136-321) STAT3b {Mouse (Mus musculus)}
>d1uura1 a.47.1.1 (A:242-359) STAT homologue coiled coil domain {Slime mold (Dictyostelium discoideum)}
>d1fioa_ a.47.2.1 (A:) Sso1 {Baker's yeast (Saccharomyces cerevisiae)}
>d1hs7a_ a.47.2.1 (A:) Vam3p N-terminal domain {Baker's yeast (Saccharomyces cerevisiae)}
>d1o5ha_ a.191.1.1 (A:) Hypothetical protein TM1560 {Thermotoga maritima}
>d1iqpa1 a.80.1.1 (A:233-327) Replication factor C {Archaeon Pyrococcus furiosus}
>d1h12a_ a.102.1.2 (A:) Endo-1,4-beta-xylanase {Pseudoalteromonas haloplanktis}
>d1fp3a_ a.102.1.3 (A:) N-acyl-D-glucosamine 2-epimerase {Pig (Sus scrofa)}
>d1lf6a1 a.102.1.5 (A:288-684) Bacterial glucoamylase, C-terminal domain {Thermoanaerobacterium thermosaccharolyticum}
>d1qaza_ a.102.3.1 (A:) Alginate lyase A1-III {Sphingomonas sp., A1}
>d2sqca1 a.102.4.2 (A:8-36,A:308-630) Squalene-hopene cyclase {Alicyclobacillus acidocaldarius}
>d1qgra_ a.118.1.1 (A:) Importin beta {Human (Homo sapiens)}
>d1qbkb_ a.118.1.1 (B:) Karyopherin beta2 {Human (Homo sapiens)}
>d1ee4a_ a.118.1.1 (A:) Karyopherin alpha {Baker's yeast (Saccharomyces cerevisiae)}
>d1gw5a_ a.118.1.10 (A:) Adaptin alpha C subunit N-terminal fragment {Mouse (Mus musculus)}
>d1b3ua_ a.118.1.2 (A:) Constant regulatory domain of protein phosphatase 2a, pr65alpha {Human (Homo sapiens)}
>d1uw4b_ a.118.1.14 (B:) Regulator of nonsense transcripts 2, UPF2 {Human (Homo sapiens)}
>d1h6ka1 a.118.1.14 (A:27-290) CBP80, 80KDa nuclear cap-binding protein {Human (Homo sapiens)}
>d1hs6a1 a.118.1.7 (A:461-610) Leukotriene A4 hydrolase C-terminal domain {Human (Homo sapiens)}
>d1b89a_ a.118.1.3 (A:) Clathrin heavy chain proximal leg segment {Cow (Bos taurus)}
>d1rz4a2 a.118.1.18 (A:2-131) Eukaryotic translation initiation factor 3 subunit 12, eIF3k, N-terminal domain {Human (Homo sapiens)}
>d1qsaa1 a.118.5.1 (A:1-450) 70 KDa soluble lytic transglycosylase (SLT70), superhelical domain {Escherichia coli}
>d1klxa_ a.118.18.1 (A:) Cysteine rich protein B (HcpB) {Helicobacter pylori}
>d1ouva_ a.118.18.1 (A:) Cysteine rich protein C (HcpC) {Helicobacter pylori}
>d1dcea1 a.118.6.1 (A:1-241,A:351-443) Rab geranylgeranyltransferase alpha-subunit, N-terminal domain {Rat (Rattus norvegicus)}
>d1ld8a_ a.118.6.1 (A:) Protein farnesyltransferase alpha-subunit {Human (Homo sapiens)}
>d1vdua_ a.118.20.1 (A:) Hypothetical protein ST1625 {Archaeon Sulfolobus tokodaii}
>d1dtoa_ b.91.1.1 (A:) E2 regulatory, transactivation domain {Human papillomavirus type 16}
>d1ciy_3 f.1.3.1 (33-255) delta-Endotoxin (insectocide), N-terminal domain {Bacillus thuringiensis, CRYIA (A)}
>d1i5pa3 f.1.3.1 (A:1-263) delta-Endotoxin (insectocide), N-terminal domain {Bacillus thuringiensis subsp. kurstaki, CRY2AA}
