# Supplementary material for: TPRpred: a tool for prediction of TPR-, PPR- and SEL1-like repeats from protein sequences
Source: BMC Bioinformatics. 2007 Jan 3;8:2. doi: 10.1186/1471-2105-8-2 (PMC1774580; doi:10.1186/1471-2105-8-2)
Supplement: Additional File 3 — STAND proteins. The set of 56 STAND family members. [file 1471-2105-8-2-S3.PDF]

>gi|77457134|ref|YP_346639.1| ATP-dependent transcriptional regulator, MalT related, LuxR family [Pseudomonas fluorescens PfO-1]
MTAMTHAPDRPGFLPRLSAHHLPRPRLTASLLESSARVRLICAPAGSGKSTLFTECLLQAPLECTVCWLPLAGLSMSVAA
FCERLAQALGLAAADESSLLNELARRSSPVWVFLDDYCRVEEPALDALLDRMLALASPAVTWWIGTRRRPQCNWPRLLLD
DAVYECERAALALSRGEIAQLLRHLPPAQAGDVAGRILQRSGGWCAGVRILLMQKCDWSQKTLPQQRMDTLLDYLQHELF
SGLSPELAEAWRVLAHLPRFNASLCDHLFGAGEGAQWMRTLQTLGCFIEPWQGSPDWLQVCAPLSRLMRDEPWSAGRSWH
RLACQWFAAGQHWKCAFEQALLAEEYEVAVSLLQHFTFEHLFEEQTVVLLLRLYEQRGEELLLGSAQLVGLITAALLFAG
RFAQAADCIGQLSRFLPPPSASQQRQLIARWQAQQGWLLHLQGHMDEARAHFEQALNDLSPQAWTARLLCLSGRTQQALL
CGDLELAHSINREALCLARAEGSLLFEALLELDHAQLLEQRGATARAEDLLAGLCEMLGASVERPTPMLGRIALRRGRLA
LTMGQQGRAAVFFQQGLDDCLRSYDKRVLYGFLGQAQLAADQGDYPRAFMRLRDAERLMQQRQIPDTVYRGVLLQVSSEF
WLQQGRPELAREALSRVLRHYRGPRARQAPPATLELVPRIECLLILSETRLHQMQTSLVRLRALLTSAQARGMVRLEAEL
LLTLTEVALILGEPDQARSFFDQGCQIITRCSLQQMLAELYIRCVEMPSRWRIDEAAEQVDEQSTTINPLSQRELKVLQL
IAFGDSNLQIAEKLFISLHTVKTHVRRIHSKLGVERRTQAVAKAKLLNLC
>gi|15600782|ref|NP_232412.1| malT regulatory protein [Vibrio cholerae O1 biovar eltor str. N16961]
MRFKLKEHEALTGIDERKKMWIPSKLTRPGRLHNAIVRPRVLDLLQHATCYKLVLFRSPAGYGKTTMAAQWLADKPNLGW
YSIDDSDNDPFRFMNYLLQAINKATHNACPNAQKLAEKRQFSSLHSLFSEVFAEMADYHGECYVVLDDYHLIHDETIHEA
MRFFLKHMPDNLTLVVTSRSTPPLGTANLRVRDLMIEIGNELLAFDTEETTRFFNQRVSDGIDALTANHLRDYVEGWPSA
MQLIALQAQHQHRTLAQTIESVSHFNHAHLWDYLVEEVFDLLDDETRYFLMQCSVLDHFDDALVSSLTGRDDALAMIESL
NRFGLFISPLEGETNWYRFHNLFAEFLAHQRQARIPQQEQDLQRAAAKAWLEAAAPHQALRHAHLAQDTELLASILSQYG
WKMFNQGELEVLEAAINQLSPPQLYREPKLCMLQAWLAQSQHRYNDVGALLAKAAKEMKALNVELSTKEQGEFNALRAQV
AINQNEPEKALELAELALSQLDHTTYRSRIVATSVVGEVNHVLGHLSRALSMMQQTEKLARQYQVYHQALWALLQQSEIL
LAQGYVQAAYEVQDNAFKLIEEQQLHQVPLHEFLLRIRAQILWCWNRLDEAEQAAYKGLSVLENHSQSKHLHCYSMLARI
AIGRGELDKAGRFIEQIQHLLKQSTYHVDWTANASLSLLLYWQVKENSTEIRQWLQSSTRPDKACNHFSQLQWRNIARAQ
IQLGELSEARHTLDFIQEQAQEYQLVTDTNRNLIVEALLAITEGDDLQACHKLKQALRLTNQTGMIGNFLIDGSKIGHLL
EKLVHKGELGDLERHRAHLLLKEISTTQRSRSIHFDEEFVEKLVNHPNIPELVRTSPLTQREWQVLGLIYSGFSNEQIAH
ELDVAGTTIKTHIRNLYQKLNIANRKEAVQTAEQLLQLMGY
>gi|504484|gb|AAC44880.1| AcoK
MKPLDLEGRQSPSLVSGPLRLTRGLPLILTKFVPPRSSIQLLERPRLLQLLSPVQQCRLGVVCAGAGFGKTTLLAQWHQQ
MVAQGERIAWLSLDEDDDDVWQFIPYLLQALRPLYADWDADFWRDMEEQKLSSSEQLLAGLINQLHYCPHDVYLIIDDFH
VINVRGVYEALGYLIKHAPAALHLIIGSRFHPNLALSQLQAQDQLVEIYDRDLQFTLEETKHYFSRTVALPLSNHHAQRL
QSVTEGWIAGMKIASLSAELQNDPEHLLRNMHGGTRSIARYLKEVVLDPLPEEVLDFLVKTSFLSRLNAELCNAVTGRDD
SKAMLAWIERHNLFLSALDEQGYWFRYHPLLQENLRTMLQQNNDIDRKQLHELASHWFVEQKLWSEAVRHALSAGKPVHS
PVQDGASAQSLAEEGDIDTLISWMHHLPPSTDPSRIDLQINLAWALAHYFHFDESRQLLDNLDQMVLHHREDLTRSTWCK
LRVVRAICEAFAENIPESLAIVQPLLAEVPCGDTWVDGLICNILSYCHVVNQRYHDALEVQQHMPSPESPLDNLFVSVYR
AFIIAQCHLCQGDLGKAGWYAEKTLRQAECYTGTQSTSGATLAPLLAEIAYECQRGDSPEHLLADRLEFIDRFSPPDALS
RCYTYLARQALDNDMPYEAERLLEHAQRLAVSRGWQRLQAMLLAEQVRVRLQRGNFTGAEQLQRQLEQMAASFRMDAEHP
CQRAIVMSASLSRSRLLLARGQAPQACVLLAEMVSDQESRGDRLTAARLRTLWSLALWNSGKTAAARTTFQPVVQLAEQQ
HLTGLFLDAGDTLQPLLAGMNETSSACKEKGGVHEPWADKRAPADGTPFNSGSPDIPGELSEREFQILQLIAEGQMNKEI
ARSLAISAETVKWHIKNIYAKLKVNSRTQAMSRALEMKLLD
>gi|48781464|ref|ZP_00278072.1| COG2909: ATP-dependent transcriptional regulator [Burkholderia fungorum LB400]
MARLLEARRQRCVVIQGPAGSAKTSTMLALRQAMLTLDFDVAWLSLAPEDDDLTAFFHGLLASLASVDPAIVREAELLVG
RESEETAVEHWVITLVRGITRRKRELVLMLDDVHYLQNARILDALRWLLDYAPPQLHVVLASRRALPVSLARLRSQGLAT
ELDREDLRFSAEESERFLREQLGDITKRDAQVVHELTDGWAAGLQLFAIDLKTRKGAAFAPATVRDAETFAHYFEREVLV
RLAAEDLLLLTRAALCERFCARLCASLMQHASSAGDVAARLSRLEGDNLFITQTGVGQDGETWYRLHPLLREVLQRRVAG
LPAAELRELHAAAWQWLSVQGYVDEAVHHAVLADNAQAAADLVEGCAHDLLARGELRQLAGLVRRLPPAQIAERFDLRVI
TAYLQMYAGETDALMQSIQQIASRSERPDPRQRYALELLRGGLALQRDDTDSVVALLPELQRVPADADDFIRAGRGNILS
WMFIHRGDYQQARKVLEEGAPHGGAPRGILLGRCMSGMTHAAEGQVLLAERVFCDVLAEAEKQGPAYVGIACMAAALLGE
ALYELNDVDASLRLLAERIGVLERVSIPDTVLRALVVLARAHRFAGRRSEAYDYLERLEDYAKRHGVDRLLANALCLRSR
WLLEEGRLDEAEAALLRCETLAARYTGAVRSTAWEISVCADLARIRMCLHLNDFDGAMSRLKPLSALSEAGGRWCRVIGL
RLQMSIAEQGRHNDRAAREHLVEALRLGHRLGLMRSLLDELGPCRQILDALPLEQDFDPVLAFYAQRLIDAGQASAPAAP
PAAAAHVPIQSLSERENEVMGLLALALPNKKIARVMDVSLDTVKWHLKNIYRKLEVSGRDGAVAKMRDASAR
>gi|68556571|ref|ZP_00595913.1| regulatory protein, LuxR:Bacterial regulatory protein, LuxR [Ralstonia metallidurans CH34]
MSQPFSAPAESASIDVFADISPDVSPDVSPDVSPLSAARAHPRGTARVVARERLLAQLTEARRRRCIVIQGPAGFGKTAV
LVAWRLDLRALGFDMAWVTLADDDNEMTHWLDHVLSSLAQVSPAITREAALLGGLGDLESEAIERTVIALVGGIAACPRE
IVLVIDELHRLTNPRVLEAMQWLLDYAPPNLHVVLASRSQVPLSLGRLRDQDLVLELDQRDLRLTADESRRFLATQLTGI
DARDARVLHELTDGWVAGLQLFATHWKRKKQDANSLSFANDFVRANVLNAGAFSAYFEREVLSRLAPDEAELLVHAAACE
RFCASLCVALTGGRADSEPVVSALLARLDNENLFIEPVQGPDRAPWYRLHPLLRETLAERFRQRSESVRHQVHMAAWQWF
RDHRLLVEAVRHAVLAGESGTAADLVERYSSTLIGRGEVRKVAGLLHQLPKPEVRARLALRLLTVQMQLYAHEVDAARAD
LDALDGELPAEGELRQRLRQLQTMWMVLTDNAEGAQRLQPLLESMPDDIGGLMVGARNNLLTWLYMHQGNYERARAIQSE
NAPLIVEGAPLLGSAAGMLNGRCMAAFSYAIEGKITQVERICRDVIRESEQAGAFGTEPEYFAAAMLGEVLYEQNQIEAA
LAMLEPRVDVLERISIPDSVLRVFMTLANCHAARGHHLDAFAYLERLEEHATRRRLDRLLAHSLAGQVRLHRRAGDPAAA
DACLARLEAIAERHRDAGPGTMDEILLVARRARVKGYLDDGDPAKAAAAVGPLIDQCQRRGWQRHLAQMQWMAALADHRL
GNDASARERVLAALRAGHRLGLVRSLLDVDPGALDEIAALAQQPGEAPDPLLAFYISRLRATGATNPMTSARAAAPATLR
TLAEPLSEREADVVELLGQALPNKKIARTLGLSPETVKWHLRNIFRKLGVTSRDEAVARVRDLSRPS
>gi|73537679|ref|YP_298046.1| regulatory protein, LuxR [Ralstonia eutropha JMP134]
MRHQIPSQARRPQRQREATDAALPRAQPGTQAPASVAPQPTSTCQMTSVNRGAGAAFSELALKTMPPRAPRHLLARARLS
LDSEALRDRPAIVVQAPPGFGKTLLLAQWRREYLAHGAAVAWISADTHDDPLHFLQCLVLAVRAGGARPGFGRTLLEGAA
AAPGSTEGLTAWLAEVAQTSLDLVLIVDEAERLSQDGVAALLYLLHNAPPNLRIVVAARGGFDTAVAELTAYGQCAGIGA

QALRFRFDETLTLVRNRFGDRVDADRCARLHELTDGWPLGLQLVLAAMERGADPRSVVDTMAARPADEGEHLVGGLIAKL
TPDDVAFLTRIAVVDHVHPDLCRALTGDEQAPGHLARLIRETPIFVSSDGSDWCRLHALARGALQARLAEVPRNEQAELH
ARALHWLAERGITEEAARHAHAAGQHALAYELAGRSLYDAVMQGRLGTVLEWLERLPDAELDRYPGLRLAAAWVLALGER
HAEAEALVARMLEGAGDDTVLRYECALILSIAAYYADQPDRCLALLEPWQQQPATHDPRLAQMHANRMAMAAILHGQTGN
ARRHLQLAPRGDLGEGHRYAARWGEFVTGLSYLREGQMMLSEAILRPTLASAEADLGRRHPLSCMLASLLAAAVYENGRV
DEAAALLANRLDVLERSGTAETSLLAYLTAARVAALQGVEHRALDLLEALYAVGVARRTPRLCMASLADQVRMHAGRFRA
ETCRALIVRLDELVAGAEADHGPLWRRDVVLMQAVAHANAAIAAQDWSSALDALRRAAPAAEAMKLGRKRIEIMALEAFV
RHRAGEDSRTLMLEAMNLAQTYGLARTFADAHPALADWARRLVEEETTEPGAGQPLPVARTPRRAERAVTEPRAVASTVL
TPKEREVLELLARNLSNKEIAQAIGVGEVTAKWHVKNLFGKLSAGTRKHAVRRAQLLGLLEGLE
>gi|47572956|ref|ZP_00242997.1| COG2909: ATP-dependent transcriptional regulator
[Rubrivivax gelatinosus PM1]
MPPSSPHRQLRSHPLEATTDWPTPLADELASTERGFPSDVRMHKLFTPPVYPGAVPRQAILDRVLQDDSLRVTVLQGPAG
HGKSTTLQQIKTAHEARGWRTAWLTLDDADNDPRRFESHLVAVMSLLHGRAASPGASRSGTGDAPRDLADWMLDTLSGRI
TPASIFIDEFQALRNEALLRFFRSVLARLPANVHVFIGSRTLPEIGLATLMVNRVASVVRADDLRFTPGEVTQFFADSAS
LQVSAGEVDAIYRRTEGWPAGVQLFRLALVSPEVRMALDGADDHGPRELAEYLADNVMSLQSPRMQEFLLKTSLLQRLSA
PLCTAVTGFEDAQELLVRLERSGLFLRALDSDNRWFRYHGLFSTYLAETLQRNGPEALRQVHKKAAQWCLAHELPEEAIH
HALCCRNFPLAASTLTDWSSQLVAGAELITLERWHDRLPFHEVAQRPALVIRAAYALMFLRRRPKLRPLLELMAPQAGGG
DIVPTTNPDLCRAMSFLLVDDDMAAAADTVEQAGVVQRELEGFPAFELGAAANVLALGKVASGDFEGARQALALARAHLG
RGGGSFVGGYTAAITGSNLLVQGRLQEALAHLRDENAQEAPLDTSVAGAALAACHMFALYEANDLATLESLAHRFQREIS
ESVTLDFIAAAHIAISRMHEARGRSDEAVAVLDELERIGHTSPWQRLVAVSEWERVRRALAGGEIERAVALATRIAPDSR
DDAPHWIHLAEDVEGSGYGWIRLAIARHDHADAAQRIARERARQTGRVYRDIKLSVLETLLQQRMGARNAAHRCLRKALQ
LGRRGRYVRCLLDEGDGVIELLREAYQNLLRGHEPGGGTGTDPDRDYIELLLEASGTDLGRQAAGNALTEALSEREKEML
RFLLDGTTNREIAGRLFVSENTVKFHLKNIYSKLGVGNRLQAINTARALRLID
>gi|78063892|ref|YP_373800.1| ATP-dependent transcriptional regulator, MalT-like, LuxR
family [Burkholderia sp. 383]
MTSSDSRQAAGPADADGIEVLVRTKLAPASARGIVSRIARFGRLARGLDRRLTLVCAPAGYGKTTVLAEWRHALVATNVK
VAWVSLDQDDDNASIFSSYVVAAIVDATGGVGGRAQRLLRDRALIPLKIVFDELLNELEVAGVELVLMLDDYDRLASPVI
HDAMFELLRYAPSNLHVVLSCRSAPALPLSYFESRDELVRVDEDDLRFDEAETRAFFERVAGKPLSAENVGRLRAATEGW
VSGLQLAALALRDDNDAARVAEQVSHARAGIAAYLNENVMTQVPDAIRHFLLCTAVLDRMTPALCDALTGRDDAVACLEW
LSAHNLFIRSVDGGRRSYRYHALFLQYLREELALRRPDAVAALHRRASTWYAAERQWPDAVRHALAAGDFDAAAGWVEAC
AMKLIAASDVRTVLDWVSRLPEPALAGRLRLRIAHVWALALSMQMVDARRALEAIEADIDARRLGTDAITATELLGVRSL
IAGLSDRSIESLELGERVLAAGPPAGSWVEQIGQTTLIFGFGYAGRLDDALDLRARAERAASGHEPLFAHVYRQNMSGLA
EFVAGRLHDASKTFETALRGAERSAGRLSAATALSAGYLSAIYYEWNDWPKVREAQRDRFDIAMQACSLGPLLRFMQTAA
VLQFRSGNEARAHEMLDEADHIACSRQWLRLRVACMATGVRLHVSAGKLTQAHRVGRALAALVSAEPPEVASSFVETWQM
AQSARARLLLADARAEEAAGVMATVHDVLAARGFDWFAAQAAVLRAVALEQAGQEDDALAVLAPALEYGQGNGLVRTFVD
EGAVAERLTARVLKRADRFPAVGAWYLRELARAFEADRASNAAPAARPGTAGNLSAREVEILDYVARGLSNKEIARALRV
APETIKWHLKNIFEKLNVTSRIQAVRSGLALDASSRTRVGDE
>gi|32041835|ref|ZP_00139418.1| COG2909: ATP-dependent transcriptional regulator
[Pseudomonas aeruginosa UCBPP-PA14]
MGHRDGVPASTIPLLQTKLFPPSAGGLPLLPRQALIDRLYEARGRRAMVLSAPAGFGKSTILCQLRLRLLEQGAAVAWLS
CDETDSEPQRLIQYLLASIQRVVPAFGGNTANLLGTDVAVPLEGILDAFLADLRRLEGPLYLFLDDFHRIRHAALPHGVR
YLIENLPDHVRVVASTRFRPRFLVDEPTLKPWTFCLSAEDLRLSREESDAFLLDLKGLELGERELKLLFKRTEGWITALH
LAALALSRNTDREGFLRGLSGTERNIADYLAEDVLASLPADLQLFLDQTSVLDEFNAELCNALTGRRDGLDMLMRLQNEQ
LFVIALDDQREWFRYHHLFAEFLQGRLSRHADPTPLLHAAARWCEGRDLADRAIKYALRARDYLFAAELLERQGARLIAG
NRVYGILGMLNGIPAEVIREHPVFQIFYAWQLAFEQRFAEAEALIEEVSHRLLQGRGKVMHFGLGELLAAAQVLKALVLL
YQDKLEACLKVARHWLSMVPSNQPVFRASLLCVQASAHALLGDYAEAAGAIGSAREDLKVADSEYLQVVTSLIESLICKE
SGDLERGRAIAESARNRVEQVFGRRSRVGGPLSLAYADLLYEQDRHAGVLAELPLATVWRDVATPVELLSRGQLVMAKAR
FFSGAAEQGLAQLDEWLSGLLSPGYERVYAHAMSCKVQFLLWLRRPNEAERVCLQLERHLAGLSGERHADAQTALVLAEA
RLALSERHAERAQSKLESCLAGYSSAYQRDRRLRLSLLLSVAYWQKGNSEKAIGLFLATLEEAWNLGYRRLFQDDALWLL
PMWESWREAEPKRAAAWQGLADLLREQCRKLSVDPESFDENQDVSHREREILRLVAAGLSNRDIAQAVHLSEATIKWHLH
NLFAKLGVKSRTQAVLKGKSLGLLSEA
>gi|73537995|ref|YP_298362.1| regulatory protein, LuxR:Tetratricopeptide TPR_4 [Ralstonia
eutropha JMP134]
MRTSAVQSAAHGALPRGSRQPASSVLPMPERTDTPPPPSTLSQAATMPARAGMAQRGAARVVARERLLAQLTEARRRRCI
VIQGPAGCGKSAALMAWRLELLGLGFDMAWLTVEPEDNDLARWLDRLLGCLSDVSPAITREAMLLAGRGLDDEGVERTII
ALVRGIAACPRELTLVLDDLHHITELRVRDAMQWLVDYAPPNLHLVLVSRAALPLSLGRLRDQGLLLELDQRDLRFTAEE
SRQFLAAQLGDIAPRDARMLHELTDGWVAGLQLFATHWKRKRQHDSGISFATDLVRANVQNAGAFAGYFEREVLSRLAPD
EAELLVRAAACERFSASLCAALSEGSQTEAEVLTLLARLESENLFIAPVEDSGRETWYRLHPLLRQTLAERFRARSERQQ
GGVHTAAWQWFRDHRMLEEAVRHAILAGEAGTAADFVERFATTLMARGEIRKALVLLRLLPAEQIQQRIELRMLTLRMHI
LMHDADAAAAIADRIEVDLPADADKLRYRLALQRLSIMVLRDDTAGAQALLPQVLQPPEQLEPFYAGGRNNLLSWLYMHL
GEFELARRVQTDAQPLMVEGAPLVGTASGSLNGRCMIGFSYALEGKMLQAERVCRDVLREVEHAGSARSEPEYFAAVLLG
ETLYEQGDLEAARRLLEDRVDVLERVSIPDTVLRVHTVLAAAHWLSGHRLDAFAQLERMEEYGNQFRLDRLVAQSLGYQV
LLHVVSGDIDTAEAVLSRLRAVALRHEHAPRGVLDAIHLSAERASILVSLAHNELDDAWRRVMGLLAYCEARGWQRHATQ
QHILAAVIASRRGDTETAQDHAMIALRLGHKLGLVRALMDTYPGSLDLLVAAAANEPQDPVLGFYVERLQAAAQVLPQMA
SGNTESTDHALAGQEELSDREADVVRLLAQALPNKKIARTLGLSPETVKWHLRNIFRKLNVSSRDEAVARMRDRGLGQDT
>gi|74016775|ref|ZP_00687400.1| regulatory protein, LuxR [Burkholderia ambifaria AMMD]
MGRLRSSCFGTHAIDQAAARPHSPRGGLTRERGSTDPSPHAHPGRWPARHRRIPTAAPMAHPPDRADAPPTELVLKTTAP
RVPAQLLARARLSLAAPAFDARPVMLVQAPAGYGKTSLLAQWRRESLALGRAVVWLSADERDDAPRLLQGLVQAVRTGCA
RPGFGRLIAGGAARALEGLTAWLAEVAQLSIDALLIVDDAERLPAAGLDALTYVLHNAPQNLRVIVAGRRGLDRAAGDLL
AGGQCTLAGPEWLRFTLDETIALVGARFAQRVDADACARLFERVDGWPLGLQLAMAAMARSADPRQAVDALAARMDGTRD
RLVELLLLANLSADDTAFLTRTSIADRLHPSLCAALLDDAEAPDRLARLARDTPLFIASDDSDWCRLHPLVRDTLRDRLAA

GPAAERSVLHGRAAAWLDAHGMTEEAARHAYAAGQVETAYALAQRCLEDAIKQGRINDVLDWLALLPDAELDKRPTLRIA
VAWALALGERHVEAQRQIAGILADAATPAAMRYECALILSAAAYYADEIDRFVELFEPWADFTPPAATWLAQMHANRLSA
RAIIVGEPAQARRYQQAAPRGGTAAGFGYVARWGELITGLSYLCEGQIRLADDALSPALARAEADLGRRHPLTCMLAAMC
AAIAYEADRVDQAAALLANRLDVLEHAGTPDTVLLGYRTSARIALLRGVEHRAIDLLEALDALGVARRLPRLSVASLAEQ
VRIHAARYREATCHALVERIDAIAAAEFPSHGPLWRRPVVLLQAIAHAGAALAARRWDDACAALDEAAVLAREMSLGGAR
IEIMALGAFALEQSGHDGRARLDEAMNLADMFGLTRTLADTHPAVADWARRIGDEAIERDAPARSAQPQPRIVPPAPRST
ASPRAVPSVVLTPKERAILELLARSLSNKEIAVALAVGEETVKWHLKNLFGKLDASSRKHVVRRALVLGLLESAP
>gi|73538584|ref|YP_298951.1| regulatory protein, LuxR [Ralstonia eutropha JMP134]
MHKGVEDIPLPARPPSRGLVASKLVPPASGPATLPRPQLVQGMLDASAARLILIRAAAGFGKTTLMQQYAVQCAARQRST
AWLRVDGGDNDLERFLVHLDAGLQALHGKRKAARGTAPADDTTGPRLAHRIIEQVASAVLPFSILLDDFETVQSASVLNF
VQQLVEAMPPCGTLVIGSRVTPEIGLGRIRARGHLLEIHPAQLRFTLEEATALIRERCHLPLRDSEIATLHRCTEGWATA
IYLATLSLQTRTDHAAFVASFSGTNLELAEYLAEDILAQQSDACRSFLLETSVLGQLSASLCDAVTGRQDSRAMIDYLER
ANLLLFPLDGDRTWYRYHQLFASFLQHRLDLQQPGRATELHRDAARWYLEQSRPVPAVDHLLQAGLHDEALPQIARQADA
LLSAGRVRLLVRWLDPIRPEALARHPRLRLARAWALLLNRRYADALQAVESIQALGDSGGSERLAVEAETIRCVLLVMTD
QVEACRQASMVQINRLGPDDLFQYCILANSLAYSLICTHRYDDARSVLSRAIQRGADERSVFMRSIADCLEGLIDLVHGR
LGNALARFHTASTRTWNDASGDITGDKPAIDTSWSLALYENDALDEMARLLADALPYTKANGPPDSVIGCHVLSARLALL
RGDKEQWLRVLAELEQLGQQVNAERSVCSAWIERARVATLEGRLDAAEQALRAVDLYGGWEARDTAGHANDIERPSITRR
RLEIAQGQHAVALAALDEAIGAAIAHQRFWRLLKLRILRATALDGLARRDEALQEITEALRLASHEGFVRTFLDEGERIA
MLVRSWASAYQTQAAGLGIAPQFVTRLLAKLPSAPVATEAEPALVAGLSDSLTARELEVLQMLSAGLRNRAIAEKLFLSE
LTVKSHLRKINAKLGAQNRTEAVAIGRSRGLIP
>gi|74023905|ref|ZP_00694471.1| regulatory protein, LuxR [Rhodoferax ferrireducens DSM 15236]
MDGFSRANALAHKPSGAAFDSLQRYMPAFSHNDPGPMSAATPAGPSVGSAGLDNKFNPPAPMAAQVQRQGLCEAICRSAG
QLVLVSAPAGFGKTTAMVQARARLEQTGVSTVWLTLDRADNDVSRFMICLGEAVTRLGLGGPDAAAGVDSVQALARSDIP
FALFLDEFETVHEAAVLGLVREIADHLPRGGHLIIGSRSLPQLGLARLRVRGLLTEIDADRLRFSLQDALTLFEQRRQPN
VLSTEQLFRLHQKTEGWATALWLASIALDRTVDQSDFVDRFSGSNRAVADYLAEDVLARQPTHIRRFLLRTSLLRQLDAS
VCAALNPRADCVALLEQLDAEHLFLSPVTGARRTWRYHSLFADYLRTQLEREHPDDVARLHLAASGWYESKDRPVPAIDH
AIEGGDFPHAMSLLDSHADAFLEQGRMRLLSRWFASMPPSQRQAHPRLEMIAIWAACFTRGPWEAMEMLERSGASASDDP
YLRANANGIRPMLLAMQDRNEESLEVGREALRQLPTGHQFADTTLLNAMAHNLAVMGDQREAQQLLDAARREQGGSSTFN
RMYTESTEGLLDLQQGRLRQATARFRLAVDATHAISHTHTHGNALAGVYYACAVYELNQLDQAEHLLNVYLPITRDIGLP
DHMILAHAMRSRIAFINGDVDAASLAITELEYLGNQRKLPRVVAAAKLERARMLQLQGNAAAAHDELMRADDPTLWSREQ
RQRMLAHDIQYMAMARLRWEIAFGDAGACLGRLDAEKKRAAEAGRHRRLLVLRLLHALALQRTGDMPAAVTEIGTALQFA
CQEGFMRLILDEGPAVGALVQRYQSLHDSGQMRDPLLGDYLQRLLQAFGPLPTEIEATPELLGGVLEPLTRKEIRVLQLL
VEGYSNSAMAEKLFVSDSTVRTHLRNINMKFGAHSRTQAVAIARRLGLIA
>gi|76259642|ref|ZP_00767289.1| regulatory protein, LuxR [Chloroflexus aurantiacus J-10-fl]
MMPLVRTKLQPPEVRPSLLPRPRLYEQLRLALHSRLVLIAAPAGYGKTSLLAEWIVEARKEWSHIAWLSLDETDNDIRRL
LAYLLEALSNLPHERRAQLQALLSEDSEIDTTQWCAELLNDLAETAGPTVIVLDDYHLINDPLIHNAITFLIDHLPPRCR
LLIASRRDPPLPLARWRARGHLTELRSFDLAFNPEETAAFLLETMELPLSRDQIVALLESTEGWPAGLRLIALAIRDRVN
RGELIDELISGNRYVADYLIEDVFNHLSHQLQQFLLQTSILDQLCGALCDAVIGVHDETEVRTPVQSSQQILTELERNNL
FLFPLGSERVWYRYHHLFAEVLRDRLMASHSPDQVAILHRRAGAWYAAHGMLTQAVNHALQAQAFDDVVAMLEPIGLAVI
SQVGEVTLRRWLPSLPVEAFERHPRLALLRAWLATADYQVDEAAHWIAVAERALAGVASGAATPIGPIANLRGELSAVRV
RLAALQGDTDQVIAEGKQALNWLQSDYQSLRMRIAKDLGYACIAQRDLVRAEQSFSEAMVNGFHVGLPYISAMATIDYAY
TLMLRGAYTNAINACRDTIDQMMHHQRNRTVPGIGLPFLALADLHTIRHEFAGALPALAEAESAIKSGHTTSYLCLLIVM
ARVARARNDLPGALRIIRQARFMARQRRVGWVRAVLDGLESQIMIEQGDLEAAGQLITNIVATPSEFQYLPTAAFYAREH
ITRAHYEWRLAHYQDTGDCQALHDLAMELSTPSGTELWQRLSPLDAALLRGLALAAAGDDATEAILSALDLATSERIVTP
FQQAGEPLRRILNQLLSRQRLPPFGEYLLSLLDDYEPSPIAVLSTPSSDALPEPLSQREIEVLRLMAEGRSNQEIAGHLI
IAISTVKSHVNNIFSKLGVASRTQAVARGRRLGLIP
>gi|37524480|ref|NP_927824.1| positive regulator of mal regulon [Photorhabdus luminescens subsp. laumondii TTO1]
MLIPSKLSRPVRLNNTVLRERLLIRLNESSNYRLVLITSPAGYGKTTLVSQWAAGQDNLGWYSLDESDNQPERFASYLIA
ALQQATQGHCVKSEVLAQKHQYANLPALFAQLFIELSEWRRPIFLVIDDYHLIVNNMIHEAMRFFLRHQPENLTLTILSR
NLPSLGVANLRVREQLLEIDSQQLAFDYQETQQFFDKRLTTSLDADDCKRLCNEVAGWATALQLIALSARQSCDAAELSA
KRLSGINAGHLSDYLVDEVLNQVGSSTREFLLRSSILRSMNDGLIVRLTGEENGQQRLEETERQGLFIQRMDDSGEWFRF
HPLFASFLRQRCQWEMALQLPELHRAAAEGWLAQGYPGEAIHHALAAGDTAMLRDILLQHAWSLFNHSELSLLEECMNAL
PYEQLLESPRLVLLQAWLAQSQHRYSEVNTLLNQAEQALQQKQIAIEPDLLAEFDALRAQVAMNSGKPEMADKLAKQALA
ALLPQNEYSRIVATSVEGEVLHCRGELADALARMQQTEQLARRFHVHHYALWALLQQSEILLAQGYLQTAYETQNKAFEL
IREQHLEQLPMHEFLLRIRSQLLWCWSRLDEAEEAARRGLEVLSNFQPQQQLQCLTQLAKCSLTRGDIDNARRYLARCEN
LLSNGQYHRDWRTNTDKLRVIIWQSMDDKSAAIRWLAQTERPESVSNHFNQGQWRNIARAQILLGLYDEAESILDTLNQH
ARELQLISDLNRNLLLCNLLYWHIGRKSEAQSALIEALSLANRTGFISHFVIEGELMAQQLRQLIQLNTLSELELHRAQR
ILRDINRQHRHKFAHFDESFVTQLLTHPDVPELIRNSPLTQREWQVLGLIYSGYSNDQIAAELDVATTTIKTHIRNLYQK
LGIANRQEAIQQAQQLMQMMGLGV
>gi|76260736|ref|ZP_00768366.1| regulatory protein, LuxR [Chloroflexus aurantiacus J-10-fl]
MQSTSQFTLITCMPIPILQTKLHPPSLADCFVPRPRLTERIHTALNNRLTIIAAPPGFGKTTVMAAWLATNTSENVAWYS
LEEDDNDPICFFTCIAAALQTVAPVSALESALGAAVANPRELTVALLNDLFVFSDRSVVLVFDDYHHITRQPIHDALVYL
IDHLPNNIHLVFISRVDPPLPLGRWRVRGQLTEIRADDLRFTEAEAAQFLNQTMGLSLSAEAIRTLEERTEGWVAGLQLA
ALSLQKSSNPGQIIAAFAGSNRYVADYLTDEVLARQPESLRDFLLKTSILERFNAALCNHLLQCVCSESILTDLERANLF
IIPLDSDANWYRYHHLFADLLKRRLAQANPAGITDLHRRAAEWFEQNNFTLDAIRHWIAADEPERVAALMERTLSQSWGH
AELAGLMHRIEALPDTVLAKYPILSAFLGWTWLWLGYDNARTLPLLERAERNLKDEPQASYARGRFQVIRSYIARAVHND
APHAWLLAEQALQQLAEHDLLWRGFAQSNIAIVTHSMGLGLAQTERAYNEAIRLCRAAGDPTTAWIAECARVQVVRECGD

LQRSMTLNRRLIDMIERQGAPALVRGWVHINQALGYYLINDLKHAWLEVNMTQNLQTQSSGMPDVSLRLYALLTRLELLN
GNEAAARQAADDLVTLAYRGGVTNAIDWAYAVRAELMFRLKDWAAFDSYARTYQPPQQPLFFPYRLQTLLQVRYLTRQKA
WNEGRRLVAEQVRLAREAGYVEYEMELHIVSALLEQKAGNPSVAMNMLGRALEIGKANGYVRIFLEEGEEMRHLLTQIHR
LRRDDFVAELLAAFGKPAQVVQSSLIEPLTEREIEVLRLIAEGLSNPEIAEKLVLSVGTVKTHVKHIYGKLNVDDRVKAA
GMARELGLLK
>gi|59713994|ref|YP_206769.1| MalT regulatory protein [Vibrio fischeri ES114]
MLIPSKLHFPRRLHNAILRQRVLDTLENAMHYKLVLFRSPAGYGKTTMAAQWLSDKDATGWYSLDESDNDEYKFAKYFVQ
TISNATHTELSHSKALTEKRQFASLSSLFSQVFTELISFQDEAYIVLDDYHLITNDTIHLGLKFFLKHLPDNLTLVITSR
SQPPLNTANLRVRDLLIEIDNQSLAFDQSETSLFFNQRLNENIDQEAITHVREHVEGWPSALQLIALHSIQNNQSLNNSA
QVMAQLNHAHLWDYLAEEVFDHLDQETQQFLMRCAVFDHFNTTLLSKVTQRKDAQLLIENLNRFGLFINTNGEQGDWYKF
HNLFAEFLTHQRQNHLAHEEIDLQKYAAEAWLEHDNIYQALRHAKNAKDDILIANILTQHGWHLFNNGELSLLETTISDL
RPEVLFTSPRLVIIRAWLAQSQHNYLQVNDLLSEAQREMADRNIVLDRHMQGEIDALRAQVAINKNEPEAALAFAEKALE
ELETNKYRSRIVATSVIGEYHHVSGNLAQALALMQQTEKMAMTYGIYHQALWAILQQSEILMAQGFMQAAFDLQDNGFKL
IEDQDLHQLPLHEFLLRSRAQIYWFWFRFDEAEQCTWKGLDVLSPFDETKHLRGYSMLARLAVARGELDKAERYLDKCQQ
LLGQSDYHIDWKANASSAQLFYWQAKNNSEAIQYWLAQAERPETRCNHFTQLQWRNIARAQFHLGDIDTAIETLQTSQTI
AQQHKLITDINRNAILAAILLYENKQIDEAVEAMHLAIKQSNQTGIIGSFLMAGRNVLKPLGKLQQKGNVTELEKHRIEQ
LQKALSNNERSRSAHFDETFIEKVLNTPEVPELIRTSPLTHREWQVLGLIYSGFSNEQIAAEFDVAPTTIKTHIRNLYQK
LNLPNRKAAIAMADELVGMMK
>gi|56315919|emb|CAI10562.1| hypothetical protein [Azoarcus sp. EbN1]
MCLVGPARGSLPHRRIRARRPRRDAPLRRRPDPQYPRLRSDHAGPRRRGHAGDRAAKRLLEPIGTVHALNYSVTFEGAIA
LLQGEAKAARARFEGTLEAVIAGGHRYTTSSAIVAAHLAEALYELNDLDAAETLLIDYLPVIHDGCLPDHIIVAYQVLAR
IQAQRGQYAKALVTLDTLQDLGDVRSIPRISAASRIDKHRLALLALLAGDLAAARRMAPLLADASVWQSSSGFLLYAENL
DDPLVADARLALVEGSVRAVIPRLQDAICEANALNRRRRVARMQCLLAQAFDVARRRPQALETLERALLQAEPGGMLRVF
ADEPWHLHDLLQALADRKCAVSPGYLQNLRAAATRTEPVAPKGPLQDEAMRLSPKEGQILRRLAEGHSNKELARKLFVTE
NTVETHLRRIYGKLGIRSRTQAVARALELGLI
>gi|77964419|gb|ABB05800.1| ATP-dependent transcriptional regulator, MalT-like, LuxR family [Burkholderia sp. 383]
MASRRDANMKSLSRPFAAPLDMSVAAPAPFASHADTGAHLDARIAAKLSPPTAGATDISRVAPRDLVCSAQSATLTLVRA
PAGFGKTTVMTQCFARFGRLGFDTGWLTLDQGDNDPSRVLHVLNLCLLRMGGERPTSGNMPSIEDVTLAVFDRIAAHATP
FVLFVDEFEAIHDQGVIALFQEIVDRLPRHGRIVIGSRSQPHLPLGRLRTQGKLLELDAEQLRFSLAETDRMLNGQTGMA
LSVEDLDHLQRKTEGWPAALRLASLALENRDATRALIDRFSGSHEAIAEYLIEDVLGSLSEPVQAFLLRCSVLRQFSVPV
CSALNPGVDGEHMLQTLEKAGLFVVRVDAHEPAYRFHSLFAEFLRAQLVRRHPDRVPGLHLAASHWYERNGYPVPAIDHA
LDAEAFVCASTLLQRHAIDFLAQGRLRLLARWFARLPAGCVADHAEVGIVHAWTVCLTRGPGEAMALLGASGWLSASSDT
VRAHASACHVALLATLDRVEDAYDEGRCCLAALPNAHPFAHSSLVNLVANVATTMGHRDEARRLLGAAKSLGDPQSVLNR
MYSEATEGLLDLQEGHLRLATARFHLAATIAGGHEATGFTDGNAWAGLLYARALYEADRVSEAEPLLNVYVPLARDIGLP
DHAIAGYAMLARLAFQRGDIDPAFLLLAQLERLGHDRRLPRVVASAHLERARLLLLQGNAEASRSQLDLAEAAAEPWARI
EALRFPAHEVETLTIAKLRWQLHFGDPDAAARALAQARGAAAADGRRWREMKLRVLECLASLRARHERDALAAAAPLIGT
LCAEGFVRMLVDEGPLAAQLALRCQADGATRARDPLLEEYLHACIAPFDVEREAPPASVPEDGTALPAESLTRKELRVLQ
LLAQGYSNGALAEKLFVSDSTVRTHLRNINAKLNVPNRTSAVAAGRRLGLVD
>gi|68556570|ref|ZP_00595912.1| regulatory protein, LuxR [Ralstonia metallidurans CH34]
MDCSVPSHKLMPPRLGGGIAMRDALLARLHEARHAALVVLAASPGYGKTTLLAQWRQQLVGARARVGWLSLGPELDAPTR
FCAAIAASVASVVQGDVSPSSRHDPTEALIAALAAVPDELYLMLDGAEYLRDTGAWAVLQALIDARLPRLHLALATRCRP
ALRLGQLGAEGVVVEFDDESLAFTLAETRACLPPESGQAASVRLMEATRGWPAGVRMLAGGRAPDDSRAALDAYWSEVVA
PGLSVGQARLLRWLAWLDRWTPELAAEVTGVPRAAECARSLVGQGIFIGPARAHAGWHTLHPLFADWLRRSMPLAGADRL
ALHRRAVAAWLRVGSSGEALDHAMRADDGGLVAHVVREAMPAMPALSQLRALPDWIDQAGVKHVVDEPALWLAAAWACTV
AARLGEAERWLAMVAGCDDVAPQVALVRASIAFQKDDPATMDVWLARLGGHALPHPSLEHIRAALAVRCGAMLHRPEFPP
VPRLEAAPHDELVAMAHGAAALTMLLRGDARRAQAIGMPAARMAQQIHGRRSVSACVCAVPVAAALLEQDRAEEAAEMMA
CWLDLAHHSSPDVMIVATLCQARLQWLAGDQHGARATLAEAQAHAAGRGLMRVQLRLLAQRVVFAIQAGDLRHAGLLQDE
LDALAATLDASDMRAREVGTIAALSRVRVALSAGNPVGALAALEPVRSDNAGALVLIDLLFARVNAALDRPQLVVRHLES
ALAAGLRLGLLRMLRDEPGIPALLDCVQPSGDAVRDNWLGRVRAAKDQLQQPATCLPMIRRRRRDGEPEHLTPRERDIVA
LLEQSMSNKRIALVLNLSVDTVKWNLRQIYAKLGVSTRYEVILAVRNRALRTGTA
>gi|77951639|ref|ZP_00816058.1| regulatory protein, LuxR:TPR repeat [Marinobacter aquaeolei VT8]
MLLTTKFLRPTPDSRAVQRQRLDTLLQPESGKRLNLVIAPAGYGKTTLVSQWCAGNSMPTAWLSLDEQDDKPRQFWQYVI
GAFEHAGLIGLEDCKKLLARQDDDELTPAITGLLNVLAADGNAWSLVVDDYHVISNDTLHRQLAWFLDYLPPGILVTLAS
RTEPPLPLARWRVRRQVQDIHPGLLAFSEEECQEFFRSTMAMELSEDDIRSICRKTEGWVAAMQLSALAGKKEAVLPGQA
LGSPDSRLISDYVLTEVLEQLPEDLKDFLLETACCPRLCAPLCNAVRQRQDSQEKLSSLLSQNLFLIPLDNSNEWFRYHD
LFREALLQRIQSAAPSHASELQSRAIDWLLRHGHVQEGIVQIVRRNDTDHLAAVLAEHGNNLIHGGYHLPVLEWLDAIPA
HKIQDSPQLQMLRIWGLFFANRVESLDPLLLEVEDLLDRRVADSHPDAEGALALHSEISLVRSYLARTRNDEKNASDLTR
QVLKDIDQISIPLKSVTYYGLGLDYFGKGELNDAEEALESAVHYGQVERKASTVLSSGGLLAWIQYHRGDTDLALETTGR
VRQWVDEHYADPSQPRLISCWQNSALTEIYRERNQISTADGYLAPLLDHVRKGTEPGQHVVIQYVRGHLAFTRGDLETAV
EALEDAVTTGRRRREHIVFEPPAASALLARCYLSSGHAEKARACLQSTLDQPATNPLNREQNQIALARVQNALGEYQQAQ
DTLSALIPAAERNAHNRHLVEILLVYAEALAHEGRNDESDAMLEKALDRAENAGFLRLFAEESTALREMLLDFPRLRTPG
RWNRELLGMLRDLAAGQPGEASFNSSQEAYHPIDTARPGGEPVNALAEPLSQREQEVLALIHQGLANKEIAERMSVAPAT
VKAHIRNLYGKLGVSRRTEALAKAREVGLLEDVPKT
>gi|47574286|ref|ZP_00244322.1| COG2909: ATP-dependent transcriptional regulator [Rubrivivax gelatinosus PM1]
MKIAVPAGFVPLPLNHKILPPHAHRHAVERSALLARLFRDVAERVVVFQGPAGHGKTSLMLQAQAASASGGALTGWLSLD
ESDNDIRRLLGHLQALLTVLGSQSPTPPNEDYAGSAGTITPRSDWLIGRLLELGRPVALFLDDLHAITDPGALRLLRELI
TNAPDRIRWFLASRVVPEVGLPRLVVGDEALVVSAEDLRFSRAEMRHVFDADRGLSVSDTELDAIFRGTEGWPAAVQLYR
LALGSASVRHSLASGCGGAPREVADYLAENVLGQQPPPVQDFLLQTSVLSRMSAALCDDVLGRSDGQEMLASLERDGLFV

RRLESGEGWFTYHAIFANFLQEQLARTQPEHRVAVHRRAAAWFESQGQLEEALHHHLSAGDDDRACEVFDHWSDALVPDG
HFATVDRWSHRLPADAATRRPGIAVKLSWAFTFLIRHEQLAPLLPVLRANLARRLIEGDPRLALSMAAVLQDDPARALEY
VAHTEHREPTSNRFRNFELAAGHNVRGFVGMMRGEFGPALHSLAQGRSLSERSNATFTLAYSLVHSGLTLVAQGQLPEAV
EHLRGAISDRRMVLEESVSKACLACALVMALYEADQTGDALALFEQFHDMIVEASLHDYLVVCYRAVARIRDQRGELALA
LETLEEAERLSYNGRWPRAVQLIQGERVRRELVAGRVDRAQTLAARLQPESRSDTDDWVRFSEETDGPRIGRIRLDLHAG
RAAQALDEIGACLAAAQRQGRVHRQIRLQTLAALAHHSLGQTRPAHRCLQLALELAAPGRYVRMFLDEGPALAALLRAHA
NLPAAAPENGAEARALLGRLIEASGTAPAPADTPWRAAALEPLTKREHKVLAMLVDYQSNEEIASALCVSRDTVKFHVRN
IYSKLGVKTRLEAIRVVKTSDTTGR
>gi|73537441|ref|YP_297808.1| regulatory protein, LuxR:Tetratricopeptide TPR_4 [Ralstonia eutropha JMP134]
MPARIAARGKADRMRSTILPMASNEETVRRPVPGSGTGALAAKLRPPLLTPYQVERSAICDTICAAGFVRLVLVRAPAGF
GKTTTMLQCRARLEAAGVPTVWLTLDRADNDATRFLGSLEVALSPVLEGAPKRPSDHAGADAGEQALALMDRVARHHGAF
TLFLDDFEALQNPAVVGLVWQLVESLPPGARAVVGTRWVPETGLGRLRARGELLEIEPAQLRFSASETESFLRHARGLKL
EQAAISALHRRTEGWPTALWLASVSMERRAQPETFIAGFSGSNAAIADYLVEDVFHHLPEPVRDFLLRTSILDQLCGPLC
DALYQWGPQPPAVADADAPAGNSADILAWLERANLFLLPLESERYGERGTGEQSQQWYRYHSLFSSFLRGQLTQTMPQAV
AELHMAASRWYEGQGRPVPAIEHAIAAGALDHALQLLDGSAGGLLAQGRMRLLTRWLEMVPPAELARWPKLQIARAWAVS
FTRGPAEAIALLQASSTEDATGELLAHVRALRHMLLNMMDRFDEARAFARNELPPLPMGYAFPDAILATSMARLAAVSGD
YVEARRLLAVARQAVRGSDSNFNKIFSESVEGLIDLRQGRLQQALGRFRIAARTMLPNRFGPTNGNAMACILLAEGLYET
GDSDRSGRLLNVYLPLSRDLGLADQIITGHLVLARIAFERGEVDQAHEWLAALEHLGHHRGLTRLVMSAMLERARLALRQ
GNRHAAEEALQRAGDPALWRTRPGVSSFASDVEDITVGRLRLALHVEPTAQVREAVERELAAAVSNQLMRRALKLRILLA
QAYQRTGDGAQALVVMAEALRFGAAEGFVRIFADEGDEVRRLVAQACARQGGNLPSAYVERLLGACGQGPDVAPSGQAAP
APLVEPLTPKEQKVLQLLAEGFSNVAMAQRLFVSETTVRTHLRNISAKLHASNRTQAVAIARQLGLL
>gi|77954021|ref|ZP_00818422.1| regulatory protein, LuxR [Marinobacter aquaeolei VT8]
MKSFKEGCAANDEFQTANSGLQSGELVRDLMEHRLRVPSVPGDTLARPNLDRIICQALQPGRLLQLEAPGGYGKTHALVS
ALASAPDVNVRWISLNAGDNAPSRLLSILAIALGLAEVLPPNGGTFEDHMTMLLVNRGRQAREAREVLVLDNVHYLTNPA
VSGLLHQLVTHLPSGLSVAMASRLSVPFESHTLELNGNNTRLGPDTLEFSRSETFEFFAKARSDSLITSVAIDHLFGLTE
GWPTPLALYRRELKQGGERRALHETPSVERFLKDCIAAQLGSAQLKSLRAIAEMDSCSDELFFAIEPSVSESGMPPSEAA
DRGLPLKPVPGRGRWYRLNPLLQAWLQTPVMGGYITRMLMASRWFEQRQQFPEALKYAVLAGDSDEVVRIASESTEALLL
GQDTASLLSLRKNLPAELLERSARLRIVYGWVHAIGGQFQQAKQLLEDFNDRDRKEHEARIYALKAFILRGEGFVQPALA
MAEKALAKGGMSTQAQLVTQIVRSSALCAAGCFQEARDANRIASKLAREAGDSGSEALAVYAHARIELGKGALRHAEQLL
RTGLDTAMQELSRPARIGETRLQLNLVLVLWHQGRLTEADRLLVNCARHAEQTRDLGLLLAMALRVLMCRAQGRMDDAFV
WIGRAERTMHTWQVDESLYLPVLEALKASCWLAQGQQESAMQAIAKLEPYRKGGCVPELFPMMPGLLDCLQVRADMANGD
LVRAREHLDAVSEKYEGGMPWGLQLHAGLLDAVLLEAEKGLPAARKRLESVVAQASREHFISPFAELMKELGPLMDKCYR
HLDNGDFTAALGRLFGCEPVAPAADPLAEPISDREQGVLELIAQGLSNQEIADKLHISLHTVKTHARRINAKLDVKSRTQ
AIVRARELGLL
>gi|78062060|ref|YP_371968.1| ATP-dependent transcriptional regulator, MalT-like, LuxR family [Burkholderia sp. 383]
MIVTVSRNAIQRSVRHDGVDLREHKFSPPAARRGAIVRHALLERVTRLGVPHVVILHAPLGSGKSTLLRQIMEIGTAKRW
TIGWLTLDENDNDPRRFETYFIALVTQMVADAGGPAPEQSPATTDNMIDWVLDRFGQIPGPIGLCIDDLQWIRDPAILRF
LRDLLRALPGRCRIYIGSRNLPDIGVSTLLVAEEALVLRMEDLRFSPAESVEFMTASDDEALDAEIASFVQARTEGWPAG
LQLFKLALARGNPWHTVDEMRDCGPLELVRYLSENTLSLQPPDAQVFLLKTSLLRRLNGSLCAEVTGAHHADAVLQRLEQ
DGLFLEALDGNPGWYRYHSLFARFLSERLGQDDPDGVLDVHRRAAHWYVRHGVPEEAMYHAVEAREYALAVRTLDEWASR
LIAGAELATVAYWFDRLPLNEVLGNRSLAIKVAWALVFLRRGSPTHPLLSYLDETRAREQDGATHNPDVVLAMATMFNND
LRGAARLANIPVLHQPARDIFEAFELGAAANLLTFSAMATWHEEDIHHMLVLANSHNDHAQAAFSQGYTLALRCILQVTS
GQPRLATEAPRATSGTRPWPYVNRGMAAVALAASRIWACYEADALDIGERLATQFEDEIALAAVPEFIALSMVSIARIHA
ARGRMIQARDTLDTLERLSFQSQWSGVREMIAWERLYFAGRSGDAARVDALLQHVTDDASPSDPVWIPITEMLSGRLLGR
IRLALFRQQLDRAGELLKAALAITPARPLLSVKLHVLQALVQHRQGHTRLALRTLQGVRETASAGGCRRALLDEGAELVA
LLDQAPDAEMPHTAQASEQRSPTAAWYGALSQREQEILRLLCSGASNREISEKLFLSENTVKFHLKNVYLKLDVKNRAQA
ILKAQLDLR
>gi|66044246|ref|YP_234087.1| regulatory protein, LuxR [Pseudomonas syringae pv. syringae B728a]
MPKQRFLHEPAMTDLSRMQGFADGAIPALEGRFFRPPLPEGYVPRARLCQRLDAGLGGRLLLVCAPAGFGKSSLAVEFCQ
GLPDNWQNVWLGLSARDSDPGRFLERLLASLQQFFPQLGTQAMALLKMRQRHQPFAFEEWLDGLLDELAMHLMLSKPLLL
VLDDYHLAQGPVLDRCLQFLLNHLPAGLVVMVTSRQRPDWHLARLRLTRQLLEINEQDLRLTHAESMAVLERHSGSLDSE
ALQSLIQRSEGWVAGLRFWLLAASEAGDESALPQTLRGSEVLIREYLLEEVIDCLPADVQAFLYDTACLERFCAELCDAA
RDGHDSAGMLGYLQAHQVFLVPLDEQGHWFRYHHLFSDLLRARQAAGAPPTRLHLNACRWFSAQGQLDEAVEQALRAGHL
DVAANLVQNLSEEQLLAEQNVGMLLRWKMDLPDDLLTSTPRLIVLYAWALGLACQLDAAEELANQLSRFLPAPSATAQKS
MLAQWLALSGIIARGRGDSEKTQHYCTEALLSLPEKRYGQRLVCLSTLANLAVANGDLWRARVLNRDALELAQRVANPLF
EALAHYDRARVLQARGEILRALEEVRQGQQRLKDLSTVRLYAVRARLTLYEGYLLTLRLQVDEGRVLLLAGLAEARACRD
ISVLIGHCVIATMEGCTGRFAEAFAELAEAERLMHIWDVPPIYYLAMITLVKCELWLLQGRMDLAEAWLLRLTQTYNGGL
GAAAPECHPQLPQHIELQRAVLERQQGDGVACEQRLQALERRAQEVGAKLLGLIAMTQQIGLLLSQARRDEARALLLRSL
PAASGGALMPFNALLGEHPEWLHEQLVQLPPCRVQAALLEKLPACCTPSPEAAHGGDCLSVRELGVLQLIAQGCSNQEIS
EQLFISLHTVKTHASHINSKLGVERRTQAVARAKTLGLLG
>gi|67156504|ref|ZP_00418097.1| regulatory protein, LuxR [Azotobacter vinelandii AvOP]
MDRRHSERRFAPVYSQGTCGLPLIATKFVPPRSPGLLLVRPRLMARLEPLAERSLALVCAGAGFGKTTLLAQWRGQLQEH
GQAVAWLSLDEGDDVPEYFCHYLLDALLPLHPGLGWRLGFLAAGLPSIDLVFGHLINAVQAVEAPLYLIVDDYHAIRNPA
IHRAFAYLLEHAPANFHVLLGSRSLPTLPLSRLRSRNQLIEVDTNDLRFNLCEARHYFADSAALTLSNAQTQRLLELTEG
WITGMQMATLSPSIKDNPAQAIDSLSHGGRLVGRYLEDVVFEPLPAAVDDFLMRTSILDRFCAELCDAVTGRHDGHAMLD
WIERHNLFVAALDDHGQWFRYHHLFAETLQARLRRRTDLDIAALHERAGNWFAGQSLWAEATRHALAAGKVESAPGRAGL
GAQSLAERGDIDTLLGWLQQLPITADEHRISLQLNLAWALAHRFRFDESRRLLGELRRWFAVHPARRDLRVKLDAVAAIC
EAFAENVQTGIALAEPLLAQIPCGDSWVDGLVCNVLSYCHLVLGQHEEAQAVQRHTLYPGAPTENLFVTVHRAFILGQSH

LRQGDLRTAEACFLQAMESADRLTGPQSSGSATLAASLAELAYEHGDWERLDALVRQRGLQIDRVAPLDGVLSVYRALSR
RALAEDDGVQARLLLQHGQEIAAQRRWNRLQAALLAEQVRLQLQRDDLAGAEHMLRRIEMLKSDEGTIPGCRHYARIARA
RLLLAQGLGLAAADCLAGVVAEFEARGQLLEAARLRPLWAIALWQGDERDAAVATLLPALQLGQRENLLCSLTDAGRALL
PLLSRCASAEAGSGELAGYLAILRERLGGDDCAEPPFSLSERESQTLRLVAAGQSNKEIARTLDISVETVKWHLKNLYGK
LQVTSRTRAMSRARELSLLE
>gi|56476561|ref|YP_158150.1| putative regulatory protein, LuxR [Azoarcus sp. EbN1]
MRNKTTEAIPYRFALKTMPPHSAKQALAPARLLERLRETAEQPLVVVTSLAGFGKTSLLVQWRRELLATGAAVAWLTVDA
SDDSATFIPALVASIRVAVGLDVPARAFDQLGQSGADLWIASEVLVQIHELARPTYVLIDDLHQLSDEHAVEFVYYLIRN
APPNFHVVATSRADPPFPVEELNAHGLYTQFVTEDLRLRLDDTIGFLRRRLGEDIDIETCARLHERTEGWPMALQIVTAA
MARKSDIAGTVAGLSGATGDIARYFSQFVLESLEPDAVAMLVRASILKTQHPELCAALSGVADSGEILSRLEQNTGLVTS
VGGDGDNYRMHPLFIEYLHSLLDALPAEELRVLHATAAEWFAAHNMLEQAADHAFVAGMRTQAMDWIEKRLRHLGVQGRI
VEVLAWLDRLPPEEITRREGIQLTAAWACALCYRPQDAERLADVILARPGVTPEIALQANIVRSAVAIHCDDYTRARGCI
ESYDPELGPLYCNTLSFVAIHTGFPDRARYYQQISDGRGRGVRNFYDAMYGAFAVGLSYLIEGQASEAAGVFRAALERAE
ASTGWRSLPAAVQAAGLAAACWELGAEDEARALLANRLDLIEQAALPDAVMLAYLTLARYENQKGREGKAFDALNSLAAI
GELRGQPRLVVASLGEQLRQHAIRNRIVSCRMLLGAIDEIVAGADRPDHGLEAELRLVREIAATRVALLDFNDEGAVLAL
ERAGGIAARLRRGRDQLTVKLLAACCEGSGDDAAARLFAEALSLAESFRLVRVIADDWPAALECLPELDRSGLAKGAGIT
PAFVERAAERCRFGWVPEATGQKTADPKNRPAQLSAREMEILGALSLGRSNKEIAKMLDVGAETIKWHMKNLLAKLNAAN
RRHAVDRARLLGIIE
>gi|9949559|gb|AAG06808.1| probable transcriptional regulator [Pseudomonas aeruginosa PAO1]
MTEPTELPAALPRLPPQHLPRPRLHQPLLRGECRLRLLCAPAGSGKTVLLGECARRAPPGVQVVWLALGGEALDPATFRR
RLAGALGLPEDSDEAQLCRRLRNASALWLLLDDYPRHPDPALDACLDRLLSAASPRVGWWLASRRRPQCNLARLLLEGEL
LEVDGGLLAFDADEVAELLRLHGRSADAATAGTLLERSGGWCAALRLRLLVGEETAGPLLQEYLQHELLDELPPPLADAA
RALAWLPQVGLELFRRLFEELPHGLDDLLARGFPLLADANQRYSLPPAIREAWREAPQRPQRDFHREACEWFAEHGETRE
AVDQALAADEPETAAGLLQKLTEEQLLHGHNIGMVLALRDELPAALLASTPRLVILNAWTLLYAGRLAEAEDCIGQLARF
LPMPSASRQRVLLAQWQGLFGILLHCRGERGAADYLREALEQLPEDAWSQGLICRSALMQLAMIEGRMDQARLIGRDALR
LAREHDSLIFEALIELERAQWLEQRGELLRAEGVLDRAQRYLEDLGQQGSPMLGRIALRRARLCLQQGREVEAGHWYRLG
LEQARENLDPWALYGYLGLALLEAGQGDLDAAFNRLLEVERLMQQRHVPDPLYRGALLLVSSALTLQQGRPAQAREILLR
VRAYFQPGRARLSPPSEPELEARVEHQLALAELYSGEAAAAEARLRGLLAILEAQGRQTLLCEVRMGIAECQFLSGQLPQ
AQQTLRSGLELAERLGLQLPQRRLRDRQPQLFAALPGEEEEGLSPLSCRELAVLGLIAQGCSNQEIGEQLFISLHTVKTH
ARRINGKLGVARRTQAVARAKALGLLA
>gi|46916437|emb|CAG23202.1| putative malT regulatory protein [Photobacterium profundum SS9]
MWIPSKLTRPARLHNAILRSRLLESLDQAPYYRLVLFRSPAGYGKTTMAAQWLNKHSHTGWFNVDESDNDTFRFANYLLQ
SINKATNNACLKTQAMAERRQFACLTTLFSELFGELSDYGEQTYLVLDDYHVINNEEIHEGMRFFLKHMPDCVTLVVTSR
TQPPLGTANLRVRDLLIEVDNHLLAFDQEETERFFHKRVSSEIETTVLNSLREQVEGWPSALQLIALHAQQKPNNLAESA
LSMANFNRGHLWDYLAEEVFDQLDRETQQFLLQCSVLDIFNAELVSDLTGRSDALSMLESLNRFGLFLNTLEGADNWYRF
HNLFAEFLRHQRYSQIPQQRTELHNNAAKAWLKQNRPQQALMHAMKSKNEDLTIEILTDYGWNMYHHGELKLLEDSISAL
STEKLFSAPRLTLLRAWLAQSQHRYDDVGELIAESEEEMKQRNLVLDDGLQGEFNALRAQVAINQSKPVDALVLSEQALG
QLPSTTYRSRIVATSVVGEVHHCLGNLSRALPMMQQTEKMARQYHVYHQALWALLQQSEILVAQGYVQAAFELLDQAYRL
VKEQHLQQVPLHEFLLRLRAQILWCWNRLDEAEECAHKSLEVLAPFDDTKSLHAYSMLARIALSRGEIDKAARYLELCNS
LMERADYHIDWRANTDLSNLFYWQIKGEKDNIANWLHQAERPDSACNHFQQLQWRNIARACIILEDYHQAEEIFAMLEDN
CKAHNLITDSNRNLVIQSILCKKQGQRKIALEHLKQALTLTNSTGMIGNFLCDANEICDLLQELVDTKQLNELELHRVQQ
LLREMTSKERNRSVHFDENFVEKLLNLPDVPELIRTSPLTQREWQVLGLIYAGFSNEQIASELDVASTTIKTHIRNLYQK
LNIANRQQAINTAENLLKLMGF
>gi|73537667|ref|YP_298034.1| regulatory protein, LuxR [Ralstonia eutropha JMP134]
MRIASRIVLSETERTELEALSAATAGNLRLAQRARMILLAAAGWQNKDIAAQVGVGRVQVARWRDRYAASRLAGIEQDLP
RGAPPVRTDVARLVALARDGGPGALSTRQLAAELGVSPASVSRHWRASGLPPRRGVALADKQPASGGVTVRAAEIVGLYI
AEPEHAIVVALEADPGPAGMAPALDAAARNSATYRRTLAASFMTALKVIHSGTATAFQAGRASGWQGFLHALAEHQPAGR
PLLILADNPVSHNDPEVRDVIAGQPWTVSFAAGQAAWMRAVQSLLRDADGLPAGIPQVLAVIGEHARGPFQWIRSAGDAG
YAQGQGAVPRALTAQAAFAPSTFAGKSAGRSFEAFTGTEFDSVATLPSAEPGLPRQFSALPAQPFLSLVPALDTRPGPMR
PLQPVASAKLLPPRHARKLLPREGLMNRLLDARRQRCVVIQGQAGAGKTSTLMAWRKALISLGFDVSWLSLAAEDNEPTR
FFDCLLASLAEIDPAMARDAAMQVGGAEDDDAIEQWVIALVQALGPRERELVLMIDDLHHITDARIYRALQRLLDYAPPH
LHLAFSSRSALSLALEALRAQGLLTEIDMRDLRFTAEESARFLEEQLGTIAPRDAAALHALTDGWVAGLQLFAVDLRARR
SADYPLTKVRDPRTFAAFFEREVLGLLAPDDLELLTRMSTCHRFCADLCTAMPGETETPSRIGDRLARLEADNLFITQVD
THDRDVWYRIHPLLRETLLARLETDKDDAADAVDMSDPDTAETPNDMADASRANDEPCAHGTARELHAAAWRWFDRRGHL
DDAVYHAVRAGDADAAATMVEGCGHALLKRGELPQLLGLVRRLPREQLQNRFGLLALVSFLQLYMRDVDGLGRSLERLDP
LCDRSNAVHQYVVCLLRAGHAVQVDDPDTVLGMLPALWAIPPEADDLWWTARRNVLSWLFILRGEFDEARRLQDDTERRS
SVPRSSLFGRYITAMSLVMEGEIERAGRSAREVLRESERQGATFLGLTCMAAGLLADILYELNDHEGACQLLEPRIGMLE
RVSLPDVVLRALTLLSNAHWLAGRRTQASACLDRLEAYAARNNLDRLQTEALTLRLRRHLQQAETERANTVFRQVQQLAQ
RYAGETGTVARHIALAAARAEVEMSLYTQDYATAAARLEALLAREAGAKPLRIAALWLQLALARHATGNTRGARPAFLSA
LRMGHGAGLIRTLLDVTGGAPQVFLELAGEGIDEPVLAFYARRLQAAAVASGAAISRPADEAAPIAVLSEREREILGLLA
QAMSNKKIASVLNVSPETVKWHLKNIYAKLGVNGRGRAAARLRDIATQERGSVLAA
>gi|68553916|ref|ZP_00593261.1| regulatory protein, LuxR [Ralstonia metallidurans CH34]
MSSYQSGQTPLASIHSHVFEPIAGTKLVPPRPARRPLAREALLARLLEARHQRCVVIQGPAGCGKTSTMVSWRQVLVSLG
FEVAWLSLAEEDDEPGRFFDCLLASIGLVEGEIARKAANLIGDGSDASRVEHWVVTLIEAVASQPRDLVLVLDDVHHIQD
ARIWQALQWLLDYAPPHLHLVLCSRGELSLSLARLHSQGLVTKFDLRDLRFSPEESEAYLRDQLGGISPRDARRLHDLTD
GWVAGLQLFALDIKAKQGAGFSPTALRDASAFAEYFEREVLQKLAEDDQKLLTCVAICSRFCAPLCAALVGEPHAVARMT
TRLVRLDSSNLFIIQVSGHEHESWYRLHPLLREVLLARVEAMPVAQQQALHAAASRWFEQRDQVEEAVRHAVLAGEANAA
ADAIEARASELLARGGLGQLTSLLRMLPPEQVESRFSLQLMQAHINLRARRLDAAAQAIGRLAQQATLGLRERYAVTVL
RGGMALMQDDIGAAAVLLPELERVPADADDFLLTARGNVVGWLHMHRGEHERARQVLADSTLRGGAMVSGLVGRCLSGLS

LLMEGKILQAEPVLRQVLNEAEQLGGEYVGAAHMAAVLLSETFYELNEIDSVCELLENRIEQLEAVSVPDAVLRALLMLA
LSHRVAGRRLEAAAYLDRLEDYAVRHHLDRLLALALAVRSRWQLQEGEIDLAEATVARAQKLSTEAGAGRASVWEIDVVA
QLSASGLALHRHDYDGAMARLRPLLDSVELAARGRHFATVLVQCAVAEDGRGNARAAREHLCGALRHGQRFGLVRSLLDA
ATGGQGLMRRLLDEQGLDPVVAFYARRLLEASSRWRGRAVAPASAVRRSTPIRTLSEREAEVLKLIAQAMSNKMVARTLS
ISPETVKWHLKNIYIKLGVSGRDEAVALLRDLAADTPDLGAPRPR
>gi|77633453|ref|ZP_00795583.1| COG2909: ATP-dependent transcriptional regulator
[Yersinia pestis Angola]
MLIPSKLSRPVRLQNTVVRDRLLVKLSSAANYRLTLINCPAGYGKTTLIAQWAADQSNLGWYSLDESDNQSERFATYLIA
AIQLATGGHCSKSEALSQKHQYANLSALFSQLFIELSNWDGPLYLVIDDYHLITNDAIHEAMRFFLRHQPENLTLIILSR
TLPSLGIANLRVRDQLLELGMQQLAFNHHEAQQFFECRLSSPLEQGDSSRLCDEVEGWVTALQLIALSSRQPNSSAQKSA
KRLAGLNASHLSDYLVDEVLDQVDSKARAFLLRCSVLRSMNDALIVRLTGEDNGQQLLEELERQGLFIHRMDDSAEWFCF
HPLFATFLRQRCQWELALELPELHHAAAEGWMALGYPAEAIHHALAAGDVGMLRDILLQHAWSLFHHSELALLEQCLTAL
PYPLLVQNPELALLQAWLAQSQHRYSEVNTLLEQAELAMQERKIPVDEILRAEFGALRAQVAINAGKPDEAEKLATDALK
YLPMAHYYSRIVATSVTGEVHHCKGELARALPMMQQTEQMARRHEAYHYALWALLQQSEILIAQGFLQAAYETQEKAFEL
IRESILSNCQCMSSCCGSAPRYYGHGHGWMRQKKRPVKGSRFWLTISHNSNYSVWRCWRSVHWPVAIWITPTCIFSAVKP
CNMVASITLIG
>gi|67908526|ref|ZP_00506925.1| regulatory protein, LuxR [Polaromonas sp. JS666]
MSTLSKAPIIELLRKSSMSWIVVYAYCDRTHKCNGRFAEAAQSLREAQVKFEDASSPYGLMWVRVISAASLLKAGRHRDA
LADSASALAEAPAFTPGAAGLAAMLRAIRALLLYERNQCTDAMVEVEAALPLLPHQGIIDAMIAGYVAASRLQAARGDLS
AALDVAAEGERVGLARGFARLQLTMVAERALLLLRAGDVKAAQRMASDHGLLPQGPQTNLHRDKAERLCARLDLAQGRPD
LALQWADSGIARARRSHQQHKLAELLMLRSLALARTGDTEGGHDALMESLRIAASNGYVRLYLDEGVELEGLLRRVVDKP
QSPTPAFSYARTLLLAAGAASARQDAAPAEQERPTERELQILGLLADGLSNGEVAGRLVLTEGTVKWHLHNIYAKLGVKN
RTGALREARARAWL
>gi|71066001|ref|YP_264728.1| probable transcriptional regulator, luxR family
[Psychrobacter arcticus 273-4]
MHKKEADNIRLPLILTKLNAPTDSVHLVLRPRLMSKLIENINHRVMLIHGPAGFGKTSLAMQLREFLITQEHRVAWLSLD
AQDNDPARYINYIVAAIHSVEDSIVVNTTALSESQSNYAINFILSELVNQLELLNEQMYLVLDDWHFITNQATNDALNFL
IECAPDHFHLIICSRTQPPLPIYTLHVKHQLCVIDSTELRFDETETSHFLHKSNKVNLRPEDIHTLWKKTEGWVASLQLI
LLMLRTQKSKDDFTNLFDDIGKIHSINEYFAENVLNNLPIDMLNFLLQTSILERLNDDLCNTVTQRTNSQSLLESLYKQG
MFIRPLDQERCWFQYHHLFAYFLQQRLKLQMPEKIKTLHLSAAQWFANNNQTNEAVDHAIAAKEMDRAISFIEKDSMWLV
EHSFMGTLLRLIDKIPEKDIEMSCELQLAIAWAHCLTHHPQKAQQALNFVEIALANKKYSNEANIRIEARVLQACINMYA
DRLDKIEQILPTHFDKEDNHNPWVVVVANNIQTYVLIHNYNFDDAIQLQQQVGRFHQHTRGPFSGVYGDCFAGIAYLKQC
KLDIAEQYFKKAQKKAWDKAGKHSHAAYLSGALLGQIYYERNQLDEAEALLMDSWLLGAEGGIANFYIATYCFSSRLAIV
KNNFSEAHAILDKGQLVAQALCIPRLEFLLKAELIKLYISQGNIQSAQHTMQQWNRLIIPKQASSIIEQLFEIRACAEAR
LLCRTGEDKKAIEILSTILQDNILKKRCYYEVCTRTLLAKSLNKAGRHTEAEDILLPALLKGFEQGMIRVFIDEDQNVIK
VIERLAKRCRQQHTTNATAEFNRWLLSLLALDKIQTENMDITRTYIENPSQARELAVKTLKKKEILILEMLEKGLSNKEI
ARNLNIGVNTVKWYLKAIYNTLGVSRRVQAVIEAKNLNILD
>gi|9947737|gb|AAG05148.1| probable transcriptional regulator [Pseudomonas aeruginosa
PAO1]
MDAVQDPAAGSLSRSRIVPPQVPAEYSLRPAIRNLLDRNPHSRVLMFSAPAGFGKTCALAALARERTVADQAVAWLSLAE
EDDDPARFCRQLIEALGEAVPQLGDDAQTYLQNTMRVPVVAVIESLLGDLDRYARPLLLVLDDLHLVSNPDIFTGLNRLV
QYAPPGLVLALGTRSQPALSLATWRAKGLLLEIGLEELRLGFEETREYLERSGLYLDEPCLKALYGQTEGWVVGVHLVSL
WLRQQPQVQEMALLDGDKQAVSAYLLAAVFERLPGDLQEALLALGVASQLSGDLANALTGRQDGQALLERLESMQLFLLP
LDRERQWYRFHLLFADFLRNRLRDSDPDRFKQLHFNASLWFTNHHMPTFAIEHACAAEDPEMIAALVDGCGLELINRGQL
SLIYRWRKHVPDEIAERYPILVLTDVWSRASDLSLGEANRIIDELLARWGESRGDGPMGDQYLSALAVKAVVALQKDDLE
LCIALARRVEVQLGQNAAFLEVAVLITAALAQVVRGQGDQARRLLGLAQQRNHFLEGRYLDMQLANVEVILALEQGQVKQ
AQLLIERLRQRARPLFEKSRSALALPTITEALTAYYRIELDGLEERLGWALGHVDVINPIDFYAQGQICLARTQRLLGRP
KEALASLAAMQALASRNQSWRFFAQAMAEEINLILQDSGPDRLKRAEQRLKSVDWNKMAAHYRNMAFNPVNWSLGVSRVR
LLQGRGHFSEALHEITQLRGTLQPGWHGLQRLRLDILAALSYQRLGYQERANSLLGECLINAEREGVRSLFIEEGDGIRQ
LLQQLESTERQPALQTFIRGLLGIWPGQGVRKPQDALEEGLTEREREVVCLAAQGLSNEEIGQRLSLALGTVKWHLHNIY
EKLKVRNRTQAIRRARELSLL
>gi|76259013|ref|ZP_00766665.1| transcriptional activator domain [Chloroflexus
aurantiacus J-10-fl]
MVSQAVTGEYNEYLLRPRLLPPPAPLHRIRRSRVEQRLAVSLEAPLTIVVAPPGSGKTVALAMLATHGGWPAAWCRADAS
DDPASLLRHLVAALSRVVAFDTDNPPATIDALINVLTSELDDETLLVIDDAHLIDERPDLRALIERLIGAQPPRLHLVLA
GRSEPVSPLIATARLRGEVLLIEPADLAFQAEEAMALWHQAGRSAPTDLDELISLSGGWALALRIALDATDWRRALGLRD
QPSPMLDEYLEREVFAPLPPLLRRCLQRSAGLRWIDADACSALDPSLNASQLIAELQRRRLFVTPFGERGVQFQPIIAAW
LARKAAADPEWPQLHRQAAIYFQQRGDHESALYHQIAAGDPAAVATFLPLARKLLAERRAEAVLDWMRRLALSGDESPEL
IEIQAAAFHQLNRLEAALAAYRRAERAFAAVSDRFGQARCLRGQAAIYLDTVQPAPATDLLRRALKLLPRQFGAERIELL
LMQAENWANRGRVDIGQRLERAAYRMAEAYRLTDVYQARVESLQPRLLLRSGRLREARQLLEERLWMERPLVRASAHREP
LLLLALIHALLGIGPQALAFAHRMLSEAQQSHNPVTEAIAELRLGHAYQVIARSDEQIARQHYSRGLRLLQQVSVPRTRA
EGYLGLTLLHGHAGDLARAEADAREGLLLAASAGDEWVAALILLALGSVTLMADDPRGYEWLDQAEKRFQTGRDTFGLFL
VHLWRALAALRAGRTTEVDQLVDQVIREAVEYGYESVLIGPSLFGPRDIAALVPLLLRARSMPVHRDTAMYLLRQGFPSI
AADDTVDDYHPGFTLRVQMLGAFRVWRGNQEIQAREWQREKAKQLFQLLLTMRGNWVQREQICAWLWPDADLEAAERQFK
VTLNTLNAALEPHRPPRVPPFFIRRQGLAYSFAPSFGAWIDVDEFELRASGALTSTDPDFARRSAQAALQLYRGDYLAEA
LYDPWTIEERERLLARYLATAVAYGEQLSAEGRHNEAIQIAEQVLRRDRGYEEAYQLLMRAHARAGSRSQAIRSYTRCVQ
ALREELGIEPLPETEALYHRIRQNEPI
>gi|48781484|ref|ZP_00278092.1| COG2909: ATP-dependent transcriptional regulator
[Burkholderia fungorum LB400]
MIPPRDTARVVARERLLTQLLEARRNRCVVLQGPAGCGKTSLLIAWREALLSLGFDIAWLTLAPEDNELAHFLDYLVASL
AQVDPAMCHEAALLGGRGMDDEAVERTIIALVRGIAAHSNDVVLVLDDLHHVANARNHEALQWLLDYAPANLHLVLVSRG

AVPLSLGRPRDQGLVLELDMRDLRFTAAESEAFLKAQLGEIGQREARHMHELTDGWVAGLQLFAIRLKRKKQGITDLAPS
DPLANAQLLDARGFAAYFEREVLSRLAPSEVEMLVRMASCTRVCAPLCIALIGHGQSPAEVRALLTRLENDNLFIAPIES
SGSETWYRLNPLFRETLLERFRARSELHQREVHLAAWLWFRDHRQHDDAVRHALLAGEPAAAADLVLRVARTMQIEGDLR
KLVGLIRLLPLAEVQTRIGLRLWMLHLQLYAREFDACAAAIARLHADIPESDTHSRYRLILLRAALAVQRDDTDEALSVL
PHLRQIPAHADSLTVGGRNNLLSWIHMRRGEYDEARRVQSEAPQPLVDGVPLMGTSAGILNGRCIAGFSYALEGKFIQVE
RISRDVLFEADQRGSAAAEAACFAAALLGEVLYEFNDLEAARKLLEDRVDVLERVSIPDSVLRVLTVLSAVHWAEGHHLD
AFAYLERLEEYATQYGLPRLVAHSVAEQIHRHLLSGQFDAAEAGLARLDMIAARAAGTVTDAATGTAAEIHAMTESSHIN
WCIAHEDFEGAACRLQALIPFCEARGWQRHVAHFQMQAAVVDAGRGRAEAAREAVLAALRRGHRLGLVRSLLDADPSALD
LIAAVVQGEPHDPVLSFYVSRLNAAKAASSSGAEPPAKPASRSAPPTAAETLSEREVRVVGLLAQALPNKKIARTLGISP
ETVKWHLKNIYGKLSVTSRDEAVARVRDLELGSTPVAGEETD
>gi|78062243|ref|YP_372151.1| ATP-dependent transcriptional regulator, MalT-like, LuxR
family [Burkholderia sp. 383]
MLVADVTPARRPPSLVATKVLPPRLPAGLVDRPRLVELAETAESKRLTVIQAPAGFGKTSLARLWLDCLRTRGALAGWLS
LDVGDDEPARFFHYFAQALQQACGDIGIAAGLSADASLLPPDAIVSTLINELVDVDDELYLFIDDYHLIGSPAIHAGVAY
FIDHAPSQVHVVICTRTEPPLPLARLRAHNELMEIDAAELRFSFDETQSFVRRECPGTLADATVKSLFVSTEGWAAALRI
AASVLKRGGAPGHDGAARIPSGASRPFAAYLEGMLERLPADLVDFMLRTSVLDRLSAPLCEAVTGVATSRPMLDTIVARQ
LLLQPLDQDGEWFRYHPLMAEYLRQRLATQCPDAIPDLHRRASRWYASQALWTDAVTHALAAGARDEAVALMAHCAMTLV
MRGDLLTLLGWQRQFPADLMQGQVRVSLAIAWGMALAMRFDDALAMLDAIERTPGAGVDPADIRHECQAVRSVIAALQDD
PRRAFALAQPCLDRPSTDTWTTNVVSNVVRFGHWKAGNLEALYATPWIPQSGDDRRLVFASVYRLSLLGHAEMQQMHFEL
AERYFSEAMQLGTRHCGPQSISVAICAPMLGQIRYEQGRLDEARALVIDLMPVIDVAVLLDSLLTAYKLLIRIAIARAEF
PHAYALLDRAQALAHARGWPRLTAAVLVERTRLHIAEGRLTEAAACVMQFDRLAGGDRGAQVDTSLEIDTYRALVTACVA
TARNDLQGAVAALDDGLRCASLRHSDYLALRLKTVLALVQLRGGERAAALDTFAEVLEVAEPAGIYRSIVDQGPEIGELL
QAMRDQARASSQTRDRLAYIERLLDGCRAQYQQDAQPARAAQRDMLSVRERDIVELIAHGQSNKEIARTLGIAPETVKSH
IKSLFMKLEVDKRARAVAVAQSLGLLGTRRLDS
>gi|15610217|ref|NP_217596.1| PROBABLE SERINE/THREONINE-PROTEIN KINASE TRANSCRIPTIONAL
REGULATORY PROTEIN PKNK (PROTEIN KINASE K) (STPK K) [Mycobacterium tuberculosis H37Rv]
MTDVDPHATRRDLVPNIPAELLEAGFDNVEEIGRGGFGVVYRCVQPSLDRAVAVKVLSTDLDRDNLERFLREQRAMGRLS
GHPHIVTVLQVGVLAGGRPFIVMPYHAKNSLETLIRRHGPLDWRETLSIGVKLAGALEAAHRVGTLHRDVKPGNILLTDY
GEPQLTDFGIARIAGGFETATGVIAGSPAFTAPEVLEGASPTPASDVYSLGATLFCALTGHAAYERRSGERVIAQFLRIT
SQPIPDLRKQGLPADVAAAIERAMARHPADRPATAADVGEELRDVQRRNGVSVDEMPLPVELGVERRRSPEAHAAHRHTG
GGTPTVPTPPTPATKYRPSVPTGSLVTRSRLTDILRAGGRRRLILIHAPSGFGKSTLAAQWREELSRDGAAVAWLTIDND
DNNEVWFLSHLLESIRRVRPTLAESLGHVLEEHGDDAGRYVLTSLIDEIHENDDRIAVVIDDWHRVSDSRTQAALGFLLD
NGCHHLQLIVTSWSRAGLPVGRLRIGDELAEIDSAALRFDTDEAAALLNDAGGLRLPRADVQALTTSTDGWAAALRLAAL
SLRGGGDATQLLRGLSGASDVIHEFLSENVLDTLEPELREFLLVASVTERTCGGLASALAGITNGRAMLEEAEHRGLFLQ
RTEDDPNWFRFHQMFADFLHRRLERGGSHRVAELHRRASAWFAENGYLHEAVDHALAAGDPARAVDLVEQDETNLPEQSK
MTTLLAIVQKLPTSMVVSRARLQLAIAWANILLQRPAPATGALNRFETALGRAELPEATQADLRAEADVLRAVAEVFADR
VERVDDLLAEAMSRPDTLPPRVPGTAGNTAALAAICRFEFAEVYPLLDWAAPYQEMMGPFGTVYAQCLRGMAARNRLDIV
AALQNFRTAFEVGTAVGAHSHAARLAGSLLAELLYETGDLAGAGRLMDESYLLGSEGGAVDYLAARYVIGARVKAAQGDH
EGAADRLSTGGDTAVQLGLPRLAARINNERIRLGIALPAAVAADLLAPRTIPRDNGIATMTAELDEDSAVRLLSAGDSAD
RDQACQRAGALAAAIDGTRRPLAALQAQILHIETLAATGRESDARNELAPVATKCAELGLSRLLVDAGLA
>gi|54022251|ref|YP_116493.1| putative serine/threonine protein kinase [Nocardia
farcinica IFM 10152]
MEIGRGGFGVVYRCLQTALERVVAVKVLPPDMDAESRERFLREEQAMGRLSGHPNIVDILQVDVTASGLPFIVMPYCTHG
SLEQLIHEQGPLGWADTLRVGVKLAGAIESAHRAQILHRDVKPGNVLLSSYGEPQLTDFGIARIPGGFRTSSSMITGSPA
FTAPEVLKGEEPTVRSDVYGLGATLFALLTGHAAFERQAGEKVVAQFLRITTQPVPDLREQDIPAPVAEAIERAMSPDPQ
QRPESAFEFGEMLRAAQRALGQLPDEMALLDTAVLLDDLGEVRATEPHPSIRTQFSTRARPSVTARRSWPLTLRPNVSQH
PPGATSTTPPPTAATKFRPPTPARAPVPRPRLLEVLRAGGRRRLALIHAPAGFGKSTVAAQWRAELTDQGVPVAWIGLDH
DDDNEVWFLAHLLQAIHRVRPAIGAGLDQALEERPADAVAFVISTLIDEVHAGGETIVVVIEDWHRITDPGAHRAMAALL
DNGCHHLRFVVTSRDQSGLPVNSMRVHDELVEIGSAQLRLTRAETGQILVERNGFELTDKQVEQIHRVTDGWPAAVQLVS
LALRGNPDPQRLISALPEGGHGIREYLAENVLDTLEPRMLDFLTAISVTERVNASLAAALSEDSEAELLLAQAEQRELFL
RRIEHDPEWYRMQPLFAEHLRARLERTDAAKLRALHRKASRWYAEHQFLRKSVDHALAATDLKLALDLVEGGGMDLIDGS
RLATLLGTVSKLPVQQVTSRSKLLMAVARANVNLQQSGAARSALGKLTNMLPGGSGEDETNRQRCEAAVLAAADAVTRDQ
TDGVLDQVADCLDNPDDLPAWTVSTAANVVSYVRLCTFDFDGARQIQEWAQPYHEKSKDPLGTVFGLCAQGAAAYEQLDI
PGANDYFERAWEVAHSRAGPRSHGVRVASAMLGELKYRAGDLATADRLLDESHQLVTRVGTVDFLLATFVTGARVKAVRG
DLATAAARLEEGERIAREYELPRLGAHVRAERLRLGLPVEPATESGPGPGLRRATGVAALIAEAEETAAVRAMLAARPSH
TDDAPVRRARALYGRTTEQGRPRAELDTALLLAETLAASGWVGEATAMLVPAAQVCARLGWPRPLLDAGPRVVAILRVLR
GELHLSPENADETRLPTHFLDEVLG
>gi|46164562|ref|ZP_00137359.2| COG2909: ATP-dependent transcriptional regulator
[Pseudomonas aeruginosa UCBPP-PA14]
MTDFSRTFSGTPSAVLPTANRFFRPPLPSAHVARPRLCRRLRDGLDGRLLLIAAPAGFGKSSLAIEFCESLDPRWQSLWL
GLSSRESDPGRFLERLLDGLRQYHPTLGEEALGLLKMRQRHQPFAFETWLDDLLDELAPCLDPQRPLLLVLDDYHLAQGA
VLDRCLQFLLNHLPEGLVLLVTSRQRPDWHLARLRLSRQLLELSEQDLRLTAEESGALMAASGLELDEDALDALLERSEG
WVAGLRLWLLARGDPEEQVSPGVHGADELIRDYLLEEVIERQPPEVQAFLAQTARFERFCAELCDTVREAGDSAAILDHL
QQHQVFLVPLDENGQWFRYHHLFSDLLLARPLPDFGPSAGSLHLRACGWFSRHGLLDQAVEQALRAGQPDVAASLVQNLS
EEQLLAEQNIATLLRWKMDLPDSLLASTPRLILLYGWALALACQLDAAEELAGQLARFLPAPDESAQRDLLAQWQALSGV
IARGRGDIDKAEAHCREALQDLAGERYGTRLQCLSTLSNLAVTRGDFWQARNYNRDALEFAQRYGNPLFEALVHYDRARV
LQARGEVARAEEEVRQGLERLQHLPAQRRYAVRGRLLLYRGYLRSLALQPDEARKWIKQGIEETRSCRDVSLVIGYCVLA
SLEGRLGNYAAAFARLGDVERLMHAWDIPPIYYLAAITLIKCELWLAQGQTELAGVWLQRLGGTYGGQAATPPECSPLLP
LHVELLQAGLERREGRPEEAARRLDRLARSTRENGALLPARLALGQLAALHLEQGREREALATLQQAMEGAVGGALLPLH
ELLVQQPEWLREQLQRFPACPGAQRMRALLPEPPAEPAGSAGEVLSGRELAVLELIAQGLSNQEISERLFISLHTVKSHA
RHINDKLGVERRTQAVARAKHMGLLR

```
>gi|68557156|ref|ZP_00596497.1| regulatory protein, LuxR [Ralstonia metallidurans CH34]
MSAATSRKPGDSDTQRRGLVASKLVPPRALPNHLVRSRLVDVMVDAASARLILVRAAAGFGKTTLMQQYLERCQAGQRAC
VWLRCDAADNDLQRFLLHLHTGLLRLPGLTRHQRDERSDTIAGETIAHEIIEIAASYAHPFAILLDDLEVIQNDAITEYL
QQLVRVLSPQGVLVIGTRATPDLGLGGLRARGQLLDINPAALRFSAEEAASFIRDKCGLPLPDGEIATLWRCTEGWAAAV
YLATLSLRGHPNHAAFVRSFSGTNIELAEYLAEDILMGQSESCRDFLLQTSLLGQLSAPLCDAVTGRHDSREMLSFLERN
NLFITPLDGEHQWYRYHSLFASFLQHRLQVTYPDKARALHVAASRWYLSEDRPVPAIEHLLQAGLDDEALPLIAIHTPAL
FQAGRVRLLARWLDRIDRKRLLADPHRMGLAYAWVLLLNRRYSEAMQTMAALLAADVPADQQAKIAFEVETLRCVLLAMT
DQIEACLAAGLAHLERIPPDAPFRYASVVNSQAFSLVASHRYEDARRVLSRLMPGANDVRTGFQRGIADSLEGVIDLVQG
RLSNALARFRAVAERGWSPSEVSSLRHAFLPAWATALYEANALEDCARVLAEAMPYCKGYAPPDAMIAGFILSARLALLG
GDRDRWLRMLAELEEIGRGIQLPRAICSAWLERVRVATIEGRLDAADQALRSAELHADWAASDSYGHANDIDFPDSIGWR
LAIAHGEHEQTASELADAIAVATQRQRHWRALKLRLLLAMALDGAGRQSEAFEPLTEAMRLASHEGLVRTFVDEGDRLAD
LLRRWADAREAEIVRAGIDLQFVRTVLAQIPTPLAVAGEEVNKARGAAIAEPLTTREQEVLQMLAAGHRNRVIAEKMFVS
ELTIKSHLRKINAKLGAQSRTEAVAIGRSLGLLR
>gi|76261137|ref|ZP_00768757.1| Transcriptional regulatory protein, C-
terminal:Tetratricopeptide TPR_4 [Chloroflexus aurantiacus J-10-fl]
MTELAEQVPSVAVSAPLLPLKITPPPVRSDEMLRPDLQALLAEVRLLPATVVVAPAGYGKTTLLAQWADQLIQTGAQVAW
LGLEATDQEPALLLAYLIGAIRRVIPSLGDQASRILQSISSLDRNWPLVAGALLSDIQRELQSLTVLILDDVHLVADGPI
TADLLGYLLRAAPPHLHIVMASRRPLTFAPLPRLRAEGTLLEVGANDLLLRRHEVVELLQRAGLTLSDEDIDLLLERTGG
WMLSVQLAVRTLTRHAPSQRRSYLQGLVTNQHDLFEYLASEVLSELPEPLVDRLICAALLGQVNPALLDEALNVRDSAQL
IEQAIAFGLPITVDSGSTNGERVFRFHPLWQRLLANRALTRFNRVFVRELHERFGEVLARHDQIEAALRHLAMAENPTAI
ARALREHAWPLIDTLQRESLRNWIERLPPEVRDRDPELLHMYGWSLFNTDRERALQLIGEAAEAYRRSGLPAQELRALRD
MTVLLFWADTPARFNLVCRQVIDAAGRANDTWSRGAALIGLMALLYSRGRFRAALRVARWVDRRPLSVLWQWLLAVLRAS
IYIQQGYPTEALSAIRAALELPRIDRNDLLRQSLLLLQAMALYQQGKRDDALSLAQESYTRLNDYAPGSVLVGNAALILT
LLLIEHEQFEVAATYLQRVRQIAGHLDDHFLTTRARIVEIYALFRSTRSAAATAQALTLLRQLQAKGENVERSVAGNNTV
LFSQELWMQSLLLIVLGEGGEPQRALELANELVTQMAQRNDGLFRALVHIYRAYLVAQQRPDDPAIATDLATACAICDRT
GVESLPFLPQTVMNWAITAALRIGLSSRAMIAALRQFDTTTLSVILMPLLEETFPLAVRLRSINLVGELGLVNAYGTLRG
LLKERSPQVRAAASEALERLVYRPPYRLVIRALGNFQVLRGDQEIRDRDWRSVKARHLLQLLLIERGRMLPREQIMDMLW
PGLDSESASNNLRVTVSRLIKALEPDRPEGAPTYYLLQQGDTYGFNVESDHSYDVARFVQVVEQGRRDLQLKRINEARKA
FQEAVNLYNGPFLPDSLYEDWSVVERERLELLFIEAALGLGHFL
>gi|74024089|ref|ZP_00694651.1| regulatory protein, LuxR [Rhodoferax ferrireducens DSM
15236]
MHGLTHAVDRLGPGVGQSALALLQGSVLAAPRAVVSLLVNDLEKVQGEVFVVLDDYQWIHDSAIHDAMAFLVSHVPSTVH
LVITSRDAPPLMLSRLRAHGEWLNVDANAMRFDAKETDHFLRTACAQPPTAAQIAQLYASTGGWAAALRIAALGPGGAGV
LEASASGASRAFTHLIEDLLASIPVDTVRFMAQTAVLEHLNVDLCNAVTAQEDSAELLDQLEQVNQLVEPLDSEGRWLRY
PQLLRDYLLGPLTQRLKIDQLQLHGRAAQWFARQSAWTDAVRHALAAGDGTQAIEWMVHCCMALVKTGDLMTLVGWRRQF
PAELLRSQPSVQLAVAWGLTLAMRYEDAVPLLAEIERANPLITGHADAQAQCLAVHAVAAGLQDDSARAGELARAWCELG
VQGDAWTFNVVSNVMRYVHWVSRDWAGVYEQPWEPYSFEEDRRNVFSTVYREVILGYVEMEQGRLGLAERHAREALRQAE
MYAGTQSVSAALAAPLLAMLHYEHDRLEEAAALLHPLVSLTDNTAILESVMQTYLVLSRIAWIQGQCERAFELIGHAEAI
GYNRGWDRLVGAMLMERLKRLIDENRLDEANATAVRLARLAALKATLPSGVHTDLLVFRDWGHALVALADRRASEARIAL
RRLFDLAKSERNDLRAIQTGTSLVLAHMADDDRDGAFETLREVILTAQRIGVHRSILDAGGDLQALLPRFLVSSHCNAEL
AISVRRLLAVDANEADKANKTGVAGTLTERERDVLMLVASGQSNKEVARSMNISAETVKTHLKSIFGKLGVQQREQAVVL
ARAVGLIAE
>gi|71676731|ref|ZP_00674471.1| TPR repeat:Kinesin light chain [Trichodesmium erythraeum
IMS101]
MTEEQFQEQLKARLDLIDQLLTCSSGEEQAILHANSQLLDAAFVKTMELVAAKMVEDGEKNAKWLQGLANELAEMMEAKE
KLDVQVMELYNAGKYDEAIPIAEQGVTLARQLWGESHAEVATNLNNLTLLYESQGRHSEAEPLYKQAIEIHKVALPANHP
SLATNLNNLANLYRAQGRYSEAEPLYKQAIEIFKIALPANHPSLATNLNNLAGLYESQGRYSEAEPLYKQAIEIFKIALP
ANHPSLATNLNNLANLYRAQGRYSEAEPLYKQAIEIDNIALPANHPSLARDLNNLAELYRAQGRYSEAEPLYKQAIEIHK
VALPANHPSLATNLNNLANLYRAQGRYSEAEPLYKQAIEIFKIALPANHPSLATNLNNLAGLYESQGRYSEAEPLFKKAI
EIDNIALPANHPSLATDLNNLAGLYSSQGRYSEAEPLYKQAIEIDKIALPANHPDLATHLNNLAGLYKSQGRYSEAEPLY
KQAIEIHKVALPANHPQRASGLNNLAGLYRAQGRYSEAEPLLKQAIEIYKVALPANHPFLATNLNNLAELYRAQGRYSEA
EPLYKQAIEIDNIALPANHPELATNLNNLAELYRAQGRYSEAEPLYKQAIEVDKIALPANHPSLATNLNNLAELYRAQGR
YSEAEPLYKQAIEVDKIALPANHPSLATHLNNLAVLYSAQGRYSEAEPLYKQAIEVDKIALPANHPSLATNLNNLAELYH
AQGRYSEAEPLYKQAIEIHKVALPANHPSLATNLNNLAELYRAQGRYSEAEPLYKQAIEIHKVALPANHPQRASCLNNLA
LLYASQGRYSEALKLMLKASKIEDKTIQAIFASSSESDRLAHIKQEVRPNLETFLSLVYQHFPHSPEAKQAALNLVLKRK
SLTATAMAAQNQAVFSGRYPQLQEIFPQWRRKCDQLLTLTYEVPRPEKRETHKQQLAQLQKECNELERKLTQQMPEIEIQ
QQMGDIRAVASELSEGEKLLEFVQFDVYDVQEKSWGKARYLAFVLAGGKPSEVEMIDLGEAENIDRLISTYRNSVIKWPL
PQQMNMWNNKPNSTETETPTKHYIYDSQPGKELREAVFDKLRDKLTGCSHLVLAPDGDLNLVPFQILPEDDGKTTLQDEY
NISYVSVGRELLRRRIKTKPTANPPLIMADPNFDWPQNAPDMAQQKESPAVSGLNVQPMLGTLAGITFPRVKATADLAKT
IGQQLGVKPFLGNEATADRLLKCQSPKQLIVATHGYYSQRKDKYLTLLKELLSSSQGKEVEILEKNYGLLDEEFLAFIER
YRNSENRDNKIREKLRAIAPLVKQQLKTTPVKPPVNNMDFETFKPNRHQAKIENPMMGSAVAFTGANTWLSGGQLPPEFP
TMAFAQDIAGLDLWETELVILIACESGLGDVQTGEGVFGLRRAFAVAGNQTLIMSLWSVPTLATMLLMEQFLDDSRSGMS
AGAALRKAQKYIRNITVGELQKSKLGSDILGELNLGKNLKIESKPLAHPYFWGAWICQSVSELGIRF
>gi|15026710|gb|AAK81534.1| Regulatory protein related to malT, positive regulator of mal
regulon (ATPase and HTH-type DNA-binding domain) [Clostridium acetobutylicum ATCC 824]
MNNNLRFIKTKFVIPSPRKNYIKRAKLINELNNLFDYKLIVIKAPAGSGKSTLVSSFIKEKKLSNVKWINLDESNNEIYS
FFSYLISALSPYLEDKTDALIQSISCMTKKEDILEIISFLVNEISSYEKPIILVFDDYHYIKEPFLNSTLERLIKYSPSN
IHYIFITRESLPIYLGEMRIRNEILEVNENELKFSKKECSDFIKSTLNTPLESYDINKIYTISEGWIGGIQLTSLAFKNS
KDLSIKVLNKYVVDYLTEEILKGLSNSEKDFLIKTSIMSYFNSELCNEILQIENSDEIINSLLNKNLFIITLDEEDGLFR
YHHLFKEFLNTNFNKLPDSTRKQFHLNAYTSLKNSGDISEAIIHLIKIKEYQKASSELEKNIEDTSCISLIRLIPLDALI
NSNELIIQKIFYHYSNMQIDECKSIISYLESFKKPELTALANIFKLYVYDFCNSMTLEDLNIVKTFKLTDITKAILYLNI
```

SPAFMIQGEYEKILYYSKESDKIATKYNIVFLSVFSKCLIASVLEEQGKLSDTLKVYNDLDTLIEKNKFISGFKFLVHLG
VAGVHMKKYDLENTEKFLNLSLSGLKLNYPFISRAILYNLMELYFLKGDLEKGSLTKRDLVDNYLNSHSLLPCQIHSAKL
KYTLPQGNYELEELKEYKSLVEDTINMSHIYLTPEDNVVYSRVLFILGEKEKALALLDKTLKDCRHLSILIHLVEGLIVK
ALILKEDSKVQKRELFNVLREALHYGIESEYLRAFIVEGEKILKIIKLLQNDVDITLTKKEKNFIDKIYKLVTPIKECIL
SERELEVLEVLCDGNSNKEIGSILNISLSTVKTHMINIYSKLHVSNRLQAVEKAKELNLLQR
>gi|78049092|ref|YP_365267.1| transcriptional regulator, LuxR family [Xanthomonas campestris pv. vesicatoria str. 85-10]
MLKPVALLTPRPTPSRDPVNPDWLLAGKLEPALPPPGAVVRGALLAALDQAQARPLTLLLAPPGFGKTTLLAQWHAHLST
REQSAVAWLSLDEEDADAGRFLGHLAYALQNAGADHALCAAVLHNRDHDPRDAAALLIRALRAAPRRISVILDDYDRLGS
SPVDELVLRLIKHSGGRLHLALATRRVPNLPLARLDLHAQLSRIGSTQLALDDTEAQALLGPHVTAPVADELRRYTEGWP
VALQLARLWLEGDTQRRQEVTVRFSGRSAQIAAYLAEQVVNDLDPDTRELLLRTSPLERFNAALADAVRQRQDSGRLLAR
LEHFHGLLVPLDGEREWFRYHPLFADFLQQLLDREHPGLSVQLHQRAARWFGDHQQLAEAVRHAWRGGCGDLAASYIARA
GTWQLLLTHGTHEVRALLRHFDHRTIRDTPALNLTQAHLHIKLGEFAHARLLLERFRDFPAALREPLQRDYTVVVALLRD
RLDEICGNPHGLTQIAAQAGALDEDDHLGRGMLLCICATTAIAQGAFAVAERYARNARDTMQRGGSEVGASRAMLSLGQS
LFYRGRLSDAEACYQQALSWCARTPQPDRVLEAAAQCLLAQLHYERGHHDDAADLLHPALELLEQHDGGVDVLAAGYGTA
LGLERVRDHSGRSALLLLEHIEQIAHGRKLARLSELAAAWRLMLLLEHPGNAAIDVLIARTGGESGLAHTLRSPHRWHDR
AAMGFALARWHRLAGRSSAALSILGQIEQACLANDNVCHLARARVRIALVLQQRGELVAALPYLYSALDHVALTQSWQAI
VELGLPAKAMLRSLRQHDPQTVGGTTRALTIQALLERLSGDDDPADDIFSERELEVLAQLARGYSNKQIARCLHLSENTV
KFHLKNLYRKLDARSRESALAQALQRGLLRGSGPHADQPLRPG
>gi|68553906|ref|ZP_00593251.1| regulatory protein, LuxR [Ralstonia metallidurans CH34]
MSLPSRILPKIVPPRLGSGTTIRQRLIGKLHAAGDRKLFLLTAGAGFGKTTLMAQWHHHLEQAGARVLWVTLAAGDGTLP
KFCAVLESALGRAGVSVAPAKMVTTEDSSDEFDLAAALLDALAQAPGESYLMIDDFHHVGDPATVGLVQAMADAMLPGLH
LVIASRTKPALRLGRLLAMEDVVAVDGAELLFNLDETEAFLKARPDVRLGPESAWQIHDITDGWPVGVRLASRAMCSQFP
DGKTARKCVCDSDNLDAYLSEEVIDELSPELLDFVLQVSVLRCFHADLAASVTGCADAAGYLAEILSQHMFLLPVERRDG
QRWYRFHPIFRTYLGKRRASVGIDARPLHYRAAHWFIQRGRFAEAMRSAVLSDDLGLVTQLVDRALPPPHSLAQLGVFSR
WLESVGCERLAGHPRLLSMGTWACALSGRHDLAEQWLRSVEQSGSTGTDAAAARHVLLLRALIATHRDDGAAALTALTAL
QALQVLQTDEPGSTPDALQDVELALRMRWLSVQGQHLRARDLYHSPAARSTRTGRGELALVAAATVAGVAMREGDALEAE
RIGAMVLRRARTIHGDRSLCAVTCTAVMAQAWYELDRVDEAREALLHCRTALRAASADLRLRAAQVFGRIMSLRESPQDA
LAYFTRAEVHFRAIGAHRGVACMLAEQQRIVLSYGDWRHARSMQTALDEIASTHADTDPQFAEVGAVVAMSRTRLLLAMG
LAREALIALDAARPFIGAMSNGRLLAVSDFLQTRALEALGRRAEAHACMQSALAACYRLGLHRTLIEEGEGLHGILLRAG
VGGGAALVEYVRGLMGECPVLESAGVTWPPIPIESNDEGVGGPKDDMPVPVLTRRELEVIALLEQSMSNKRIALTLNISV
QTVKWNLKNIFVKLQVTSRYEAIIVARKFALCER
>gi|75428383|ref|ZP_00731653.1| ATP-dependent transcriptional regulator [Actinobacillus succinogenes 130Z]
MKCKRREIYLRFAMKEKLKSIMLIPSKLICSYRLQNSVQRTRLTQLLNKSYLYPVVLVNAPAGYGKTTLLSQWIENIQNV
GWYSLDEGDNKTDRFAVYFSAALYTATNETDPLSEEKRQANLLALFNQLLIKVSRFPQHLYLVIDDYHLIENDEIHEALK
YWIKHQPSNMTLILISRSVPPLGIASLRVQEQLLEIDMNQLMFDHSECIEFFQTRLGSQLKQQDMIALCNEVEGWPTALQ
LISLFAKNKSRTLQVPLQDIAKRLAVSNNYHINEYLADEVLNKVDNNTRLFILRCSVLHSMNEYLVRAVTGEKNSCNKLE
SLEKQGLFLQQMANSKWQSIDDNWWKFHPLFASFLNFHCHHELRDELPELHRRAANAWVKLGYVTEALYHAMQLSDTTLL
LNLLAEHGWSLFHQGELKQLEESLNRLAYSHLSKYTNLVLLKAWLVQSQHRHTEVSGILAEFSQTLTENKIELSKTAQAE
FNVLRAQVAINSGDENTALQLASDALKDLSENAYYAHIVATSIIGEAHHCHANLPQALAMLQKAERMARQHHTYHNILWS
LLQQSEILLAQGFSQAAYEMLDKAHEFVKENHLQKVPMYEFLLRLKGKILWEWYNLDKAESMATAGMQVLQKNEDKLQCL
TLLTKISLVRGHLDNTARLLHETEQLEGTHTYHHDWIANADQIHIFYWQMTNDVESVRNWLIQNPAPVSDKNHFTQIQWR
NIARARILLGQYEQAKDILDKLIETAEAFSLISDLNRALIVRNRLYHLQGMKESAQQDLIAALKLTRQTNFISAFVIEGD
VMAQQIRNLLQLNVLDELVLHKAQFILRNINQFYRHKFAHFDEDFVNRLLNNPKVPELLKISPLTQREWQVLGLIYSGYS
NEQISDELQVAATTIKTHIRNLYQKIGVTNRNEAISYTKELLILMGYN
>gi|70728347|ref|YP_258096.1| transcriptional regulator, LuxR family [Pseudomonas fluorescens Pf-5]
MTRCPERPGFMPRLSSHHFSRSRLSAPLLASGARVKLLCAPAGSGKTALLAECLQQAPETCRVSWLPLSGGRHSPQGLCA
ALAQALGLERDDEATVLQHLAHLQAPAWIFIDDYCRVAAADLDLLLDRLLSLDNPQLTWWLGCRRRPLCNWPRLLLDDQL
FELDGPALAFSQIEIEQLLQRLPQPLCAQTASRILQRSGGWCAGVRIALLEGSELTIQPCKPGRPNTLLEYLEHELFSVL
PAELAQVWQVLAHLPRFNAELCEHLFGVGEGAQYLRTLQDMACFIEPWDGGGDWLQVFPPLARLMRDEAWPGGRSWHRRA
CQWFIAQEDWQSAFEQALRAEEYEVAVSLLEHFSFEFLFQQDNALLLLQLHERHGGELLLVSPHLVGLLVAAMLFAGRFE
QAGQCMAQMARFAPQPSAAQQRQLLARWQAQQGWLLHLSGRMAAARQHFIEALDELADSAWTTRLLCLSGLTQQALLGAE
LDVAQALNREALCLARAQDAQLFEGLLELDHAQLLEQRGAPHRAQSLLEQVCERLSMQALRATPLLGRIALRLGRLALRQ
GHAEQAAGYFRSGLEDCLLSHDKRALYGFLGQAQLAANQGDYAQAFVRLRDAERLMQQRQIPDTVYRGVLLQVSSQLWLQ
QGRPELAQEALSRLLRHFRGPQALQAPPATLELIPGIEYLLVLAEVHLGRAQAPIATLQGLLARAQRHGMQGLEAEVQLA
LAEVAHLQGNPALAQEALQAGQALVQRCNLPQALHELRLRQPLLLAAGEALQTPSSAGPLENRESLLSLRELQVLQLIAQ
GCSNQQIAEQLFISLHTVKTHGRRIHSKLGVERRTQAVAKAQALGLMS
>gi|68556956|ref|ZP_00596298.1| regulatory protein, LuxR:Bacterial regulatory protein, LuxR [Ralstonia metallidurans CH34]
MTSPPSMTRHVPSMKLRPPVAPASQVIRQGVGDMLRAASSARLVIVRAPAGFGKTTAMLQYRARLEAASVATAWLTLDNA
DNDPVRFLSGFEAALAAIVGDDAIADIGRPAPRTTGEAALQSMDCLAGYPEPFAFFLDDFEVLQEPGVIAWVREIIDHLP
HNGQLIIGSRSTPDVRLGRLRARGQLLEIDATALRFSIEETEQFFRTVRPMPLAADDLQRLYRKTEGWAAALSLASASLE
RSEAHTEFIDRFSGTSRSVADYLAEDVVARQPEPVRQFLLRTSILRYLNPQLCHVLLPDVDCEAILRQLESSSLIAPIES
EDRAWRYHSLIAEFLKAQLQREAPAEVPRLHHAAAGWFQSQDRPVPAIDHAIEGGDYAYAVTLLIAHAPALLGQGRMRLL
TRWFDALPEDVLRDHLPLQAIRIWALTFTRGPLEAMDLLERSGLRDSDDPAVRPHVLALRPTLLSMMDQLERALEVGREC
LTHWPTGVAFADMVLANAMANVFAVSGQHVESRRLLETARRTQGADSSAFNMMYSETVEAIFDLQGGRLREATARLRIAV
GSGSTQHYGYTGGNAWAGVMYAATVYEANDLAQAAHLLRVYVPLARDVLLGDHVTLGHVMLSRIAFARGDVDEAFETLTQ
LEYLGHVRRQARVAAGAKLERARILLMQGHTRAAKDELARADDPALWERVGRLHLIANDLDYLELAQLRWEALAGDAQAA
VARLRAAAEFADHDGRHRRAMKLRLLCALAYQRAGEPAAAQAILAPVLKAACAEGFARLLLDEGGNRIGPLLAQFSRTLE

SSGGAQRDPVLAEFVMRLVQACGPLEESPEPIAGDRPQLLDPLTPKEIRVLQLLAEGYSNSAIAEKLFVSDSTVRTHLRN
INNKICAHSRTQAVAIARRLGVIA
>gi|76260381|ref|ZP_00768018.1| TPR repeat:Bacterial transcriptional activator domain:Tetratricopeptide TPR_4:Tetratricopeptide TPR_4 [Chloroflexus aurantiacus J-10-fl]
MVVSAEDILAVWQWRAGGHAAHAGWRKTEWFGSTSPELHRIRRCWPCAWIVDRMPIGLPFHPGIIQGTVCPLEDCYTGMN
EETVLLQAKVAPPRPHRYRLVRPAVTARLREAFDYRVTLVQAGAGYGKTTALAELAMSSATVCWYTIGENDRDPVSFLTY
LAAACAPVLPQGAPEALATLRSHPADRAVWTQTVDGLLNALAGSPRRPVLLILDDYHFVAASAEIRALTERLITYAPPWL
SLLIATRYPIVSGELVRWRARGEVLELHRDALAFTHDEIAALFRDVFGMALSDADVDLLDHHTEGWPIALQLVWQGLRSG
QARSVSELLASGPASLAALFDFLASDVLDRQPPEIASFLRATAPLRVLTAAACDAVRQADDSDQLLAQVRDRDLFVVELD
ASHYRYHHLFHDFLRQQTRSDPDLAERHRRAARHYAAAGQAEEAIYHWFAAGEVAIAADAIVAAGEDALRNGRLDTLTDW
IDALPAELIASRPRLQSYLGDLYRLRARFDEARNWYAQAETISRQHGDRAELARALYGQALVYIDQVQPVQAESVLQEAL
RVSEGLEDQVARARVLELLAENKLNMGQPDEAEALQQQARQLRAASPAADLLSARVKLRTGQLAAARTLLQAWRDRERVA
TARGATPAPRGHREAVLVLALIAAFEGDVDQALSLAEEGVQVGTERHSRFITSVAYARLGHAWLLKSALTGVSARDRALE
YYRQALAEGQAIGVDRLRVEPLWGLTRLYGLAGDLAAAENAAVDGQAFCRWAGDLWLGAMIQLQRGVSHLLGGQFDRAQE
ILLAARADLRACSDRFGQAVATLWLALGYYEQRHDAAATAAIDEALDLSATAGYDYLFTRPTFLGLPDPRRVIPLLLATR
ARGHHRDYIERLLSAMGLRGLEAHPGYQLRVQTLGMFRVWRGDHEIEAREWQRDKARQLFQALIVHRNRWLQRDELAELL
WPQLNPEAAVRDFKVALSALYRALEPNRTEATSAFIAREGSAYRLRPTADIWLDCAEFSAGCTTGLRLLDQGQIGAGMQR
LQAALQLYQGDFLPDTIYEGWADAERERLRSMFLRSADRLAQCLAEQGQNEELIALAERILTYDNCWERAYRLLMLAYAR
QGNRAAALRVFQRCSDNLARELNVDPAPETIALAERLRHGEMPLPHEAVG
>gi|46143282|ref|ZP_00135542.2| COG2909: ATP-dependent transcriptional regulator [Actinobacillus pleuropneumoniae serovar 1 str. 4074]
MQATFSRLIPTKLISSKVNRTVKETETVERTLLLNELNQAEFYPMTLVIAPAGYGKTTLVCQWKEKQLAKNQRIGWYSLD
ESDNKTEQFSAYFTAALSQATDLSFSGIVYQNNLVDYFSQLLIQLSQVRSHFYLVIDDYHHIENSEIHDALRFWLKHQPQ
TMSLIVLSRLTPPLSITNLRIHEQLLEIDVHQLAFTPTETRQFLALKFNESLTDDEVFSLCDRVEGWATALQLVSFAVKQ
NPELLRSPEKLFAKLNQQHIADYLNEEVFHYVEPEIKLFMQRCAILRSMNEKLILALTKDENGVKKLDELEKMGLFIQRI
LHDDGEIWWKFHPILASYLAQSCRLELPCEWQELHKIAAMMWLKLGYGSEALYHAQMLEDSQTLYTILQEHGWSLFHQGQ
LKLLEDCLALLSAQQLWQDSNLVLLKAWLAQSQHRHQEVSGILQKFQPNQTLVADLQARFDALKAQVAINEGNDEQAYNL
AKQALVHLSSDFGYAQIVANSIIGEAQHCRGYLKEGLVQMQKVEKMALEQRAYHQWLWSKLQQAEILSAQGFWQSAYDLL
KDTTLQAQHLHQIPMHEFLLRLKGQILWEWHHLDQAEAMANAGIEVLEKEGEQAHCLALLAKVSLTKGDLNNAGRLIEQC
RNLIIAQAVHWDWRSTFDEVQMLYWQISDDKSHLESWLTQVSFPEQDNNHFLQRQWRNIARCYLLQDNFEQALNILNRLL
QTSATFNLISDTQRALILRNRVFYRQGRMDLAQKDLIRALNLTQQTNFISAFVIEGDLMAQQIRQLLQINVLDELSTHKA
QFILRSINQHNRHKFAHFDEEFVANLLKNPQVPELLKISLLTAREWQVLGLIYSGYSNEQISQELVVAITTIKTHIRNLY
QKIGVANRSEAIEYTRSLLRMMGYS
>gi|47572961|ref|ZP_00243002.1| COG2909: ATP-dependent transcriptional regulator [Rubrivivax gelatinosus PM1]
MEVSTHKLFAPPIYPGAVRRRVILDRVLQDNSLRVTVLQGPAGHGKSTTLQQIKTAHEARGWRTAWLTLDDADNDPRRFE
SHMRALVGLMRGGAAHPEADAAWVGDAPPDLANWMLDSLSGMETHASIFLDEFQALRNDAILRFFRFLLSRLPAHAHVFI
GSRSLPEIGLATLLVNRMATVLRADDLRFTPGEVTQFFAESKDLRVSAEEVGAIYRRTEGWPAGLQLFRLGLGSPEVRTS
LVDPEDHGPRELAEYLTDNVVTLQSPRIQEFLFKTSLLRRLSAPLCAAVTGFDDAQDILEQLERSGLFVRALDSDNRWFK
YHGLFSSFLADSLHRSSELEVVSVHERAARWYLAQHLPEEAIYHALSCRNFSLAAAILSEWSSRLISNAELITLERWYDR
LPFDHVANRPVLAIKAAYSLMFLRRRAKLRPLVDLMNQHAGCGDILQTTSPDLCRAMARLLFEDDLHAALDIIDRPEVLQ
QEVSGFPAFELGAACNVLSFGRIAAGDFEGARKSLMLARAHSERGAGSFVMGYTTAVSGARLIVQGKLNEAIERYRADML
AQHAPLDKSFAPAVMAAAQIWALYEANDLAALEPLCAQFQRGISECVTLDFIALAYVSISRMHDARGRSAEAQEVLEELE
RMGHDSHLDRLVSLADWERVRRAVLAGELDRATALAKRIAPVCPPLNPQWIYMADDMEGEAFGRIRLAIAHHDHALASQL
IARERARQTGRVYRDIRLYVFEALMLHDKGSPNGAHRSLRKALQLARPGRYVRCFLDEGRAIVSMLREEYQHFLQSAADG
QAPADPDRDFIELLLAASGTDLGQPRGRHHLTDPLSEREKEMLQFLANGVANKEIASRLFVSENTVKFHLKNIYSKLGVS
GRVQAINAARTLRLVS
>gi|68175316|ref|ZP_00548566.1| regulatory protein, LuxR:Helix-turn-helix, Fis-type [Frankia sp. CcI3]
MDTKARPTLAVSTKFQAPTYDTQLVGRDRLLDVLRDGRARRLALIHAPAGFGKTTLAVQWQRVLRAEGVPVAWLSLDRDD
NDAVWFLSHLIEAVRRVEPTLGGDLVEVLEGHSDDAQRYVLTELVNQLAEHRRPLAIVLDDWHLIDAPQAVAALEFLLEA
GPANLHLIVTSRTRSPAVGRLKVRNQVTEIDATQLRFDHRESAAFLLELNELDLDSTDVHRLWSSTDGWVAALQLATLSL
RDSADPSALIRGFSGRHHSLGDYLAENVFDALPADLLDFLLTTSVCDRLCGDLAAAVSGQRRGQALLEELERRDLFLRPL
DDNREWFRYHHLFVGYLRRRLERDHADRVVTLHRTASAWFADHGLLGEAVTHALAAGNDAGAVDLVERQAMHLVEHSRMA
VLLSLVNKLPSALLPGRSRLQIAIAWANCLLQRAQPAQVALDHVRAALTPDDTSDTGKDILGEADVVQACIDVYGDRIYR
AADLIAPYIAKNSGYRPWLVAVSSNIRTFVDIHTFAYDTAQARQRWANAFHDTTRGPFAGVYGRCFAGLAAFAQLDLVTA
ERLYREAVALAREAAGPRSHAARLAGALLGRLYYERGDIDAAERLLEECHELGAESGVADLMIATYSTLARIKTLRGEIE
DVWHLLDEGSEAASQLVLPRLSAAVDHERLRLHLARGDLGRAQNVLARQSDDVARGGDGIAMATRHYQLAMRARVMAAQA
DYDGALRLLSQMHQESSSVAWRYAETATRIDLAVVHSLTGDTDTALRTLVPAVVAGARCGLVRTVTDAGPELLKIIGELR
DASRCRRWPVELPTVPSDYLSKLLATAHADAERAAIPIIDRPAERNPAPEEPLNAREIDILRLLDRGLSNKEIARNLGLT
INTVKWYLKSIYTKLGVARRGESVSEARRRRILA
