# Supplementary material for: TPRpred: a tool for prediction of TPR-, PPR- and SEL1-like repeats from protein sequences
Source: BMC Bioinformatics. 2007 Jan 3;8:2. doi: 10.1186/1471-2105-8-2 (PMC1774580; doi:10.1186/1471-2105-8-2)
Supplement: Additional File 4 — Structure-based sequence alignments. Structure-based sequence alignments for TPR and SEL1-like repeat families. [file 1471-2105-8-2-S4.PDF]

TPR

1qqe_5a	ESNFLKSLIDAVNEGDSEQLSEHCKEFDNFMRLD
1kt0_3a	EKGLYRRGEAQLLMNEFESAKGDFEKVLEVNAAR
1e96b_3b	CEVLYNIAFMYAKKEEWKKAEEQLALATSMKSEP
1fch_1a	HPQPFEEGLRRLQEGDLPNAVLLFEAAVQQDPKH
1hxi_1a	EEAWRSLGLTQAENEKDGLAIIALNHARLDPKDX
1elr_3a	AKAYARIGNSYFKEEKYKDAIHFYNKSLAEHRTP
1a17_3	IKGYYRRAASNMALGKFRAALRDYETVVKVKPHD
1elw_3a	GKGYSRKAAALEFLNRFEEAKRTYEEGLKHEANN
1iyg_1a	RDYVFYLAVGNYRLKEYEKALKYVRGLLQTEPQN
1fch_2a	MEAWQYLGTTQAENEQELLAISALRRCLELKPDN
1fch_5a	YLLWNKLGATLANGNQSEEAVAAYRRALELQPGY
1fch_4a	PDVQCGLGVLFNLSGEYDKAVDCFTAALSVRPND
1kt1_3a	EKGLYRRGEAQLLMNEFESAKGDFEKVLEVNPQN
1ihg_2a	LSCVLNIGACKLKMSDWQGAVDSCLEALEIDPSN
1kt0_2a	LAAFLNLAMCYLKLREYTKAVECCDKALGLDSAN
1kt1_2a	LAAFLNLAMCYLKLREYTKAVECCDKALGLDSAN
1a17_1	AEELKTQANDYFKAKDYENAIKFYSQAIELNPSN
1elr_1a	ALKEKELGNDAYKKKDFDTALKHYDKAKELDPTN
1a17_2	AIYYGNRSLAYLRTECYGYALGDATRAIELDKKY
1e96b_1b	SRICFNIGCMYTILKNMTEAEKAFTRSINRDKHL
1elw_2a	HVLYSNRSAAYAKKGDYQKAYEDGCKTVDLKPDW
1elw_1a	VNELKEKGNKALSVGNIDDALQCYSEAIKLDPHN
1ihg_3a	TKALYRRAQGWQGLKEYDQALADLKKAQEIAPED
1kt0_1a	AAIVKEKGTVYFKGGKYMQAVIQYGKIVSWLEME
1kt1_1a	AAIVKEKGTVYFKGGKYVQAVIQYGKIVSWLEME
1qqe_4a	NKCFIKCADLKALDGQYIEASDIYSKLIKSSMGN
1fch_6a	IRSRYNLGISCINLGAHREAVEHFLEALNMQRKS
1ihg_1a	SEDLKNIGNTFFKSQNWEMAIKKYTKVLRYVEGS
1qqe_2a	GNTYVEAYKCFKSGGNSVNAVDSLENAIQIFTHR
1elr_2a	MTYITNQAAVYFEKGDYNKCRELCEKAIEVGREN
1qqe_1a	ADLCVQAATIYRLRKELNLAGDSFLKAADYQKKA
1e96b_2b	AVAYFQRGMLYYQTEKYDLAIKDLKEALIQLRGN
1fch_3a	QTALMALAVSFTNESLQRQACEILRDWLRYTPAY

SEL1-like repeats

1klx_1a	NGCRFLGDFYENGKYVKKDLRKAAQYYSKACGLNDQ
1klx_2a	DGCLILGYKQYAGKGVVKNEKQAVKTFEKACRLGSE
hcpc_2a	SGCFNLGVLYYQGQGVEKNLKKAASFYAKACDLNYS
hcpc_4a	DGCTILGSLYDAGRGTPKDLKKALASYDKACDLKDS
hcpc_3a	EGCASLGGIYHDGKVVTRDFKKAVEYFTKACDLNDG
hcpc_5a	PGCFNAGNMYHHGEGATKNFKEALARYSKACELENG
hcpc_6a	GGCFNLGAMQYNGEGVTRNEKQAIENFKKGCKLGAK

PPR

pfam PPR alignment was used.
