# Supplementary material for: TPRpred: a tool for prediction of TPR-, PPR- and SEL1-like repeats from protein sequences
Source: BMC Bioinformatics. 2007 Jan 3;8:2. doi: 10.1186/1471-2105-8-2 (PMC1774580; doi:10.1186/1471-2105-8-2)
Supplement: Additional File 5 — True negative data set used in servers benchmarking. Arbitrarily selected 53 true negative sequences from the all-α class of the SCOP database. [file 1471-2105-8-2-S5.PDF]

>d1hu3a_ a.118.1.14 (A:) Eukaryotic initiation factor eIF4G {Human (Homo sapiens)}
sdpeniktqelfrkvrsilnkltpqmfnqlmkqvsgltvdteerlkgvidlvfekaidep
sfsvayanmcrclvtlkvpmadkpgntvnfrklllnrcqkefekdkadddvfekkqkele
aasapeertrlhdeleeakdkarrrsignikfigelfklkmlteaimhdcvvkllknhde
esleclcrllttigkdldfekakprmdqyfnqmekivkerktssrirfmlqdvidlrlcn
wvs
>d1paqa_ a.118.1.14 (A:) Translation initiation factor eIF-2b epsilon {Baker's yeast (Saccharomyces cerevisiae)}
dfekegiatveramennhdldtallelntlrmsmnvtyhevriatitallrrvyhfiatq
tlgpkdavvkvfnqwgllfkrqafdeeeyidlmniimekiveqsfdkpdlilfsalvsly
dndiieedviykwwdnvstdprydevkkltvkwvewlqnad
>d1h6ka2 a.118.1.14 (A:291-480) CBP80, 80KDa nuclear cap-binding protein {Human (Homo sapiens)}
mfdytddpegpvmpgshsverfvieenlhciikshwkerktcaaqlvsypgknkiplnyh
ivevifaelfqlpapphidvmyttllielcklqpgslpqvlaqatemlymrldtmnttcv
drfinwfshhlsnfqfrwswedwsdclsqdpespkpkfvrevlekcmrlsyhqrildivp
ptfsalcpsn
>d1upka_ a.118.1.15 (A:) Mo25 protein {Human (Homo sapiens)}
kspadivknlkesmavlekqdisdkkaekateevsknlvamkeilygtnekepqteavaq
laqelynsgllstlvadlqlidfegkkdvaqifnnilrrqigtrtptveyictqqnilfm
llkgyespeialncgimlrecirheplakiilwseqfydffryvemstfdiasdafatfk
dlltrhkllsaefleqhydrffseyekllhsenyvtkrqslkllgellldrhnftimtky
iskpenlklmmnllrdksrniqfeafhvfkvfvanpnktqpildillknqaklieflskf
qndrtedeqfndektylvkqirdlkrpaqq
>d1utca1 a.118.1.4 (A:331-355) Clathrin heavy-chain linker domain {Rat (Rattus norvegicus)}
eeniipyitnvlqnpdlalrmavrn
>d1lrv__ a.118.1.5 (-) Leucine-rich repeat variant {Azotobacter vinelandii}
tpigdcrvcsfrmslllltgrctpgdacvavesgrqidrffrnnphlavqyladpfwerra
iavryspvealtplirdsdevvrravayrlpreqlsalmfdedrevritvadrlpleqle
qmaadrdylvrayvvqrippgrlfrfmrdedrqvrklvakrlpeeslglmtqdpepevrr
ivasrlrgddllellhdpdwtvrlaavehaslealreldepdpevrlaiagrl
>d1e7ua1 a.118.1.6 (A:525-725) Phoshoinositide 3-kinase (PI3K) helical domain {Pig (Sus scrofa)}
hpialpkhrptpdpegdrvraempnqlrkqleaiiatdplnpltaedkellwhfryeslk
dpkaypklfssvkwgqqeivaktyqllakrevwdqsaldvgltmqlldcnfsdenvraia
vqklesledddvlhyllqlvqavkfepyhdsalarfllkrglrnkrighflfwflrseia
qsrhyqqrfavileaylrgcg
>d1m8za_ a.118.1.8 (A:) Pumilio 1 {Human (Homo sapiens)}
grsrlledfrnnrypnlqlreiaghimefsqdqhgsrfiqlkleratpaerqlvfneilq
aayqlmvdvfgnyviqkffefgsleqklalaerirghvlslalqmygcrviqkalefips
dqqnemvreldghvlkcvkdqngnhvvqkciecvqpqslqfiidafkgqvfalsthpygc
rviqrilehclpdqtlpileelhqhteqlvqdqygnyviqhvlehgrpedkskivaeirg
nvlvlsqhkfasnvvekcvthasrteravlidevctmndgphsalytmmkdqyanyvvqk
midvaepgqrkivmhkirphiatlrkytygkhilaklek
>d1n4ka1 a.118.22.1 (A:436-602) IP3 receptor type 1 binding core, domain 2 {Mouse (Mus musculus)}
spaevrdldfandaskvlgsiagklekgtitqnerrsvtklledlvyfvtggtnsgqdvl
evvfskpnrerqklmreqnilkqifkllqapftdcgdgpmlrleelgdqrhapfrhicrl
cyrvlrhsqqdyrknqeyiakqfgfmqkqigydvlaedtitallhnn
>d1n8va_ a.118.21.1 (A:) Chemosensory protein Csp2 {Cabbage moth (Mamestra brassicae)}
kytdkydninldeilankrllvayvncvmergkcspegkelkehlqdaiengckkctenq
ekgayrviehlikneieiwreltakydptgnwrkkyedrak
>d1r8se_ a.118.3.1 (E:) Exchange factor ARNO {Human (Homo sapiens)}
nrkmamgrkkfnmdpkkgiqflvenellqntpeeiarflykgeglnktaigdylgereel
nlavlhafvdlheftdlnlvqalrqflwsfrlpgkaqkidrmmeafaqryclcnpgvfqs
tdtcyvlsysvimlntdlhnpnvrdkmglerfvamnrgineggdlpeellrnlydsirne
pfkiped
>d1l5ja1 a.118.15.1 (A:1-160) Aconitase B, N-terminal domain {Escherichia coli}
mleeyrkhvaeraaegiapkpldanqmaalvellknppageeeflldlltnrvppgvdea
ayvkagflaaiakgeaksplltpekaiellgtmqggynihplidalddaklapiaakals
htllmfdnfydveekakagneyakqvmqswadaewflnrp
>d1eyha_ a.118.9.1 (A:) Epsin 1 {Rat (Rattus norvegicus)}
hnyseaeikvreatsndpwgpssslmseiadltynvvafseimsmiwkrlndhgknwrhv
ykamtlmeyliktgservsqqckenmyavqtlkdfqyvdrdgkdqgvnvrekakqlvall
rdedrlreerahalktkeklaqta
>d1kpsb_ a.118.12.1 (B:) Ran-GTPase activating protein 1 (RanGAP1), C-terminal domain {Mouse (Mus musculus)}
tdlstflsfpspekllrlgpkvsvlivqqtdtsdpekvvsaflkvasvfrddasvktavl
daidalmkkafscssfnsntfltrllihmgllksedkikaipslhgplmvlnhvvrqdyf
pkalaplllafvtkpngaletcsfarhnllqtlyni

```
>d1qc7a_ a.118.14.1 (A:) FliG {Thermotoga maritima}
mfvfedilklddrsiqlvlrevdtrdlalalkgasdelkekifknmskraaallkdeley
mgpvrlkdveeaqqkiiniirrleeageiviargggeelim
>d1dlwa_ a.1.1.1 (A:) Protozoan/bacterial hemoglobin {Ciliate (Paramecium caudatum)}
slfeqlggqaavqavtaqfyaniqadatvatffngidmpnqtnktaaflcaalggpnawt
grnlkevhanmgvsnaqfttvighlrsaltgagvaaalveqtvavaetvrgdvvtv
>d1ngka_ a.1.1.1 (A:) Protozoan/bacterial hemoglobin {Mycobacterium tuberculosis, HbO}
ksfydavggaktfdaivsrfyaqvaedevlrrvypeddlagaeerlrmfleqywggprty
seqrghprlrmrhapfrislierdawlrcmhtavasidsetlddehrrelldylemaahs
lvnspf
>d1b0b__ a.1.1.2 (-) Hemoglobin I {Clam (Lucina pectinata)}
slsaaqkdnvksswakasaawgtagpeffmalfdahddvfakfsglfsgaakgtvkntpe
maaqaqsfkglvsnwvdnldnagalegqcktfaanhkargisagqleaafkvlagfmksy
ggdegawtavagalmgmirpdm
>d1h97a_ a.1.1.2 (A:) Trematode hemoglobin/myoglobin {Paramphistomum epiclitum}
tltkheqdillkelgphvdtpahivetglgayhalftahpqyishfsrleghtienvmqs
egikhyartlteaivhmlkeisndaevkkiaaqygkdhtsrkvtkdefmsgepiftkyfq
nlvkdaegkaavekflkhvfpmmaaei
>d1a6m__ a.1.1.2 (-) Myoglobin {Sperm whale (Physeter catodon)}
vlsegewqlvlhvwakveadvaghgqdilirlfkshpetlekfdrfkhlkteaemkased
lkkhgvtvltalgailkkkghheaelkplaqshatkhkipikylefiseaiihvlhsrhp
gdfgadaqgamnkalelfrkdiaakykelgy
>d2gdm__ a.1.1.2 (-) Leghemoglobin {Yellow lupin (Lupinus luteus)}
galtesqaalvkssweefnanipkhthrffilvleiapaakdlfsflkgtsevpqnnpel
qahagkvfklvyeaaiqlevtgvvvtdatlknlgsvhvskgvadahfpvvkeailktike
vvgakwseelnsawtiaydelaivikkemddaa
>d1irdb_ a.1.1.2 (B:) Hemoglobin, beta-chain {Human (Homo sapiens)}
vhltpeeksavtalwgkvnvdevggealgrllvvypwtqrffesfgdlstpdavmgnpkv
kahgkkvlgafsdglahldnlkgtfatlselhcdklhvdpenfrllgnvlvcvlahhfgk
eftppvqaayqkvvagvanalahkyh
>d1it2a_ a.1.1.2 (A:) Hagfish hemoglobin {Inshore hagfish (Eptatretus burgeri)}
piidqgplptltdgdkkainkiwpkiykeyeqyslnillrflkcfpqaqasfpkfstkks
nleqdpevkhqavvifnkvneiinsmdnqeeiikslkdlsqkhktvfkvdsiwfkelssi
fvstidggaefeklfsiicillrsay
>d1ash__ a.1.1.2 (-) Ascaris hemoglobin, domain 1 {Pig roundworm (Ascaris suum)}
anktrelcmkslehakvdtsnearqdgidlykhmfenypplrkyfksreeytaedvqndp
ffakqgqkillachvlcatyddretfnaytrelldrhardhvhmppevwtdfwklfeeyl
gkkttldeptkqawheigrefakeink
>d1itha_ a.1.1.2 (A:) Hemoglobin {Innkeeper worm (Urechis caupo)}
gltaaqikaiqdhwflnikgclqaaadsiffkyltaypgdlaffhkfssvplyglrsnpa
ykaqtltvinyldkvvdalggnagalmkakvpshdamgitpkhfgqllklvggvfqeefs
adpttvaawgdaagvlvaamk
>d1q1fa_ a.1.1.2 (A:) Neuroglobin {Mouse (Mus musculus)}
rpeselirqswrvvsrsplehgtvlfarlfalepsllplfqyngrqfsspedslsspefl
dhirkvmlvidaavtnvedlssleeyltslgrkhravgvrlssfstvgesllymlekslg
pdftpatrtawsrlygavvqamsrgwdg
>d1gvha1 a.1.1.2 (A:1-146) Flavohemoglobin, N-terminal domain {Escherichia coli}
mldaqtiatvkatipllvetgpkltahfydrmfthnpelkeifnmsnqrngdqrealfna
iaayasnienlpallpavekiaqkhtsfqikpeqynivgehllatldemfspgqevldaw
gkaygvlanvfinreaeiynenaska
>d1or4a_ a.1.1.2 (A:) Heme-based aerotactic transducer HemAT, sensor domain {Bacillus
subtilis}
etayfsdsngqqknriqltnkhadvkkqlkmvrlgdaelyvleqlqpliqenivnivdaf
yknldhesslmdiindhssvdrlkqtlkrhiqemfagviddefiekrnriasihlrigll
pkwymgafqelllsmidiyeasitnqqellkaikattkilnleqqlvle
>d1tu9a_ a.1.1.2 (A:) Hypothetical protein PA3967 {Pseudomonas aeruginosa}
naadrvmqsygrccastgffddfyrhflasspqirakfattdmtaqkhllragimnlvmy
argmsdsklralgashsraaldirpelydlwldallmavaehdrdcdaetrdawrdvmgr
giaviksyygs
>d1jboa_ a.1.1.3 (A:) Phycocyanin alpha subunit {Synechococcus elongatus}
mktpiteaiaaadtqgrflsntelqavdgrfkravasmeaaraltnnaqslidgaaqavy
qkfpytttmqgsqyastpegkakcardigyylrmvtyclvaggtgpmdeyliaglseins
tfdlspswyiealkyikanhgltgqaaveanayidyainals
>d1qgwc_ a.1.1.3 (C:) Phycoerythrin beta subunit {Cryptophite (Rhodomonas sp.), cs24}
dafsrvvtnadskaayvggadlqalkkfisegnkrldsvnsivsnascivsdavsgmice
npslispsgncytnrrmaaclrdgeiilryvsyallsgdasvledrclnglketysslgv
pansnaravsimkacavafvnntasqkklstpqgdcsglasevggyfdkvtaais
>d1qlab1 a.1.2.1 (B:107-239) Fumarate reductase {Wolinella succinogenes}
tgnwfngmsqrveswihaqkehdiskleeriepevaqevfeldrciecgcciaacgtkim
redfvgaaglnrvvrfmidphdertdedyyeligdddgvfgcmtllachdvcpknlplqs
kiaylrrkmvsvn
```

>d1fpoa1 a.2.3.1 (A:1-76) HSC20 (HSCB), N-terminal (J) domain {Escherichia coli}
mdyftlfglparyqldtqalslrfqdlqrqyhpdkfasgsqaeqlaavqqsatinqawqt
lrhplmraeyllslhg
>d1hdj__ a.2.3.1 (-) HSP40 {Human (Homo sapiens)}
mgkdyyqtlglargasdeeikrayrrqalryhpdknkepgaeekfkeiaeaydvlsdprk
reifdrygeeglkgsgc
>d1nz6a_ a.2.3.1 (A:) Auxilin J-domain {Cow (Bos taurus)}
dpeklkilewiegkernirallstmhtvlwagetkwkpvgmadlvtpeqvkkvyrkavlv
vhpdkatgqpyeqyakmifmelndawsefenqgqkply
>d1gh6a_ a.2.3.1 (A:) Large T antigen, the N-terminal J domain {Simian virus 40, Sv40}
shmreeslqlmdllglersawgniplmrkaylkkckefhpdkggdeekmkkmntlykkme
dgvkyahqpdfggfwdateiptygtdeweqwwnafneenlfcseempssddeat
>d1du2a_ a.2.4.1 (A:) Theta subunit of DNA polymerase III {Escherichia coli}
mlknlakldqtemdkvnvdlaaagvafkerynmpviaeavereqpehlrswfrerliahr
lasvnlsrlpyepklk
>d1fxkc_ a.2.5.1 (C:) Prefoldin alpha subunit {Archaeon Methanobacterium
thermoautotrophicum}
aalaeivaqlniyqsqveliqqqmeavratiseleilektlsdiqgkdgsetlvpvgags
fikaelkdtsevimsvgagvaikknfedamesiksqknelestlqkmgenlraitdimmk
lspqaeellaava
>d1fxka_ a.2.5.1 (A:) Prefoldin beta subunit {Archaeon Methanobacterium
thermoautotrophicum}
qnvqhqlaqfqqlqqqaqaisvqkqtvemqinetqkaleelsraaddaevykssgnilir
vakdelteelqekletlqlrektierqeervmkklqemqvniqeamk
>d1urfa_ a.2.6.1 (A:) Protein kinase c-like 1, pkn/prk1 {Human (Homo sapiens)}
gipatnlsrvaglekqlaielkvkqgaenmiqtysngstkdrkllltaqqmlqdsktkid
iirmqlrralqadqlenqaap
>d1eiya1 a.2.7.2 (A:6-84) Phenylalanyl-tRNA synthetase (PheRS) {Thermus thermophilus}
laaiqnardleelkalkarylgkkglltqemkglsalpleerrkrgqelnaikaaleaal
earekaleeaalkealere
>d1qoja_ a.2.9.1 (A:) C-terminal UvrC-binding domain of UvrB {Escherichia coli}
spkalqqkiheleglmmqhaqnlefeeaaqirdqlhqlrelfiaas
>d1coja1 a.2.11.1 (A:2-90) Fe superoxide dismutase (FeSOD) {Aquifex pyrophilus}
vhklepkdhlkpqnlegisneqiephfeahykgyvakyneiqekladqnfadrskanqny
seyrelkveetfnymgvvlhelyfgmltp
>d1ix9a1 a.2.11.1 (A:1-90) Mn superoxide dismutase (MnSOD) {Escherichia coli}
sytlpslpyaydalephfdkqtmeihhtkhhqtyvnnanaaleslpefanlpveelitkl
dqlpadkktvlrnnagghanhslfwkglkk
>d1us6a_ a.2.13.1 (A:) Transcriptional repressor TraM {Agrobacterium tumefaciens}
velrpligltrglpptdletitidairthrrlvekadelfqalpetyktgqacggpqhir
yieasiemhaqmsalntlysilgfipkvvvn
>d1tjla1 a.2.14.1 (A:7-110) DnaK suppressor protein DksA, alpha-hairpin domain
{Escherichia coli}
rktsslsilaiagvepyqekpgeeymneaqlahfrrileawrnqlrdevdrtvthmqdea
anfpdpvdraaqeeefslelrnrdrerklikkiektlkkveded
>d1ohzb_ a.139.1.1 (B:) Endo-1,4-beta-xylanase Y {Clostridium thermocellum}
gdvngdgtinstdltmlkrsvlraitltddakaradvdkngsinstdvlllsryll
>d1h9ea_ a.140.1.1 (A:) Thymopoietin, LAP2 {Human (Homo sapiens)}
pefledpsvltkdklkselvannvtlpageqrkdvyvqlylqhltarnrpplpagt
>d1h9fa_ a.140.1.1 (A:) Thymopoietin, LAP2 {Human (Homo sapiens)}
rqedkddldvteltnedlldqlvkygvnpgpivgttrklyekkllklreqgtesrss
>d1jeqa1 a.140.2.1 (A:559-609) DNA binding C-terminal domain of ku70 {Human (Homo
sapiens)}
yseeelkthiskgtlgkftvpmlkeacrayglksglkkqellealtkhfqd
>d1kcfa1 a.140.2.1 (A:3-38) Mitochondrial resolvase ydc2 N-terminal domain {Fission yeast
(Schizosaccharomyces pombe)}
tvklsflqhickltglsrsgrkdellrrivdspiyp
>d1h1js_ a.140.2.1 (S:) S/mar DNA-binding protein Tho1 {Baker's yeast (Saccharomyces
cerevisiae)}
gsadyssltvvqlkdlltkrnlsvgglknelvqrlikddeeskg
>d1a62_1 a.140.3.1 (1-47) Rho termination factor, N-terminal domain {Escherichia coli}
mnltelkntpvselitlgenmglenlarmrkqdiifailkqhaksge
